# Identification of patient demographic, clinical, and SARS-CoV-2 genomic factors associated with severe COVID-19 using supervised machine learning: a retrospective multicenter study

**DOI:** 10.1186/s12879-025-10450-3

**Published:** 2025-01-28

**Authors:** Kuganya Nirmalarajah, Patryk Aftanas, Shiva Barati, Emily Chien, Gloria Crowl, Amna Faheem, Lubna Farooqi, Alainna J. Jamal, Saman Khan, Jonathon D. Kotwa, Angel X. Li, Mohammad Mozafarihashjin, Jalees A. Nasir, Altynay Shigayeva, Winfield Yim, Lily Yip, Xi Zoe Zhong, Kevin Katz, Robert Kozak, Andrew G. McArthur, Nick Daneman, Finlay Maguire, Allison J. McGeer, Venkata R. Duvvuri, Samira Mubareka

**Affiliations:** 1 Sunnybrook Research Institute, Toronto, ON Canada; 2 Shared Hospital Laboratory, Toronto, ON Canada; 3 Sinai Health System, Toronto, ON Canada; 4 North York General Hospital, Toronto, ON Canada; 5 Faculty of Computer Science, Dalhousie University, Halifax, NS Canada; 6 Department of Community Health & Epidemiology, Faculty of Medicine, Dalhousie University, Halifax, NS Canada; 7 Michael G. DeGroote Institute for Infectious Disease Research, McMaster University, Hamilton, ON Canada; 8 Department of Biochemistry and Biomedical Sciences, McMaster University, Hamilton, ON Canada; 9 Public Health Ontario, 661 University Avenue, Toronto, ON Canada; 10 Department of Laboratory Medicine & Pathobiology, University of Toronto, Toronto, ON Canada; 11 Laboratory for Industrial and Applied Mathematics, Department of Mathematics and Statistics, York University, Toronto, ON Canada

**Keywords:** COVID-19, SARS-CoV-2, Machine learning, Viral genomics, Disease severity, Data integration

## Abstract

**Background:**

Drivers of COVID-19 severity are multifactorial and include multidimensional and potentially interacting factors encompassing viral determinants and host-related factors (i.e., demographics, pre-existing conditions and/or genetics), thus complicating the prediction of clinical outcomes for different severe acute respiratory syndrome coronavirus (SARS-CoV-2) variants. Although millions of SARS-CoV-2 genomes have been publicly shared in global databases, linkages with detailed clinical data are scarce. Therefore, we aimed to establish a COVID-19 patient dataset with linked clinical and viral genomic data to then examine associations between SARS-CoV-2 genomic signatures and clinical disease phenotypes.

**Methods:**

A cohort of adult patients with laboratory confirmed SARS-CoV-2 from 11 participating healthcare institutions in the Greater Toronto Area (GTA) were recruited from March 2020 to April 2022. Supervised machine learning (ML) models were developed to predict hospitalization using SARS-CoV-2 lineage-specific genomic signatures, patient demographics, symptoms, and pre-existing comorbidities. The relative importance of these features was then evaluated.

**Results:**

Complete clinical data and viral whole genome level information were obtained from 617 patients, 50.4% of whom were hospitalized. Notably, inpatients were older with a mean age of 66.67 years (SD ± 17.64 years), whereas outpatients had a mean age of 44.89 years (SD ± 16.00 years). SHapley Additive exPlanations (SHAP) analyses revealed that underlying vascular disease, underlying pulmonary disease, and fever were the most significant clinical features associated with hospitalization. In models built on the amino acid sequences of functional regions including spike, nucleocapsid, ORF3a, and ORF8 proteins, variants preceding the emergence of variants of concern (VOCs) or pre-VOC variants, were associated with hospitalization.

**Conclusions:**

Viral genomic features have limited utility in predicting hospitalization across SARS-CoV-2 diversity. Combining clinical and viral genomic datasets provides perspective on patient specific and virus-related factors that impact COVID-19 disease severity. Overall, clinical features had greater discriminatory power than viral genomic features in predicting hospitalization.

**Supplementary Information:**

The online version contains supplementary material available at 10.1186/s12879-025-10450-3.

## Background

Coronavirus disease 2019 (COVID-19) has become a significant global health concern, causing an estimated 700 million cases, 7 million deaths, and millions of reinfections over the course of the pandemic and post-pandemic periods (up to March 2024). Manifestations of COVID-19 range from asymptomatic infection to critical illness and death. Approximately 80% of cases are mild to moderate, whereas 14% and 5% of cases made up severe and critical illness respectively in the pre-vaccination era [1]. According to World Health Organization (WHO) criteria, oxygen saturation less than 94% is an indication of severe illness while critical COVID-19 is characterized by respiratory and/or multiorgan failure [1]. An increase in population level immunity has reduced the ICU-to-hospitalization ratio from 0.26 to 0.05, whereas the death-to-hospitalization ratio dropped from 0.15 to 0.06 [2].

The effects of host factors such age, sex, and pre-existing comorbidities on COVID-19 disease severity is widely understood [3–6]. Systematic review and meta-analysis of patient related risk factors repeatedly highlight that older age, male sex, underlying immunosuppression, and pre-existing conditions such as diabetes, hypertension, and malignancy were most strongly associated with severe COVID-19 [6, 7]. In addition to demographic and health related predispositions of severe COVID-19, social economical determinants expose vulnerable populations to increased risk of severe disease [6]. Racial minorities, those with poor access to healthcare, essential workers, and homeless populations all are significant risk factors for disproportionate rates of increased infection, severe disease, and mortality [6].

The causative agent of COVID-19, severe acute respiratory syndrome coronavirus-2 (SARS-CoV-2) has acquired numerous changes in its genomic sequence since its emergence, leading to the circulation of new variants. Defining features of variants of concern (VOCs) include one or more of the following: increased transmissibility or changes in COVID-19 epidemiology; OR increased virulence or changes in clinical presentation; OR reduced the effectiveness of public health measures and interventions [8]. While many differences in COVID-19 disease presentation can be attributed to host factors, viral factors are also involved in determining disease phenotype. Viral factors such as coinfection [6, 9] and genetic variation of SARS-CoV-2 have been shown to affect disease pathogenesis, clinical presentations, severity, and outcomes [6]. One of the first variants of SARS-CoV-2 bore the D614G mutation in the spike protein and was associated with increased infectivity compared to wildtype virus [10]. The subsequent Alpha VOC was associated with increased disease severity [11, 12]. Conversely, the Omicron VOC has been associated with milder symptoms and fewer hospitalizations [13]. Genomic changes can also affect the ability of the virus to transmit effectively. The Delta VOC has been reported to be more transmissible with a statistically significant increase in the pooled mean effective reproductive number, which is 97% (95% CI: 76–117) greater than that of non-VOCs [14].

In addition to studies linking VOCs to epidemiological significance and disease severity, other research has focused on identifying significant mutations within individual genes that may impact viral phenotype and disease severity. For example, missense mutations in spike and synonymous mutations in the nucleocapsid were identified in severe COVID-19 patients with underlying medical conditions [15]. Infection with a SARS-CoV-2 variant containing a 382-nucleotide deletion in ORF8 was associated with lower odds of developing hypoxia in patients requiring supplemental oxygen compared to infection the wildtype virus [16]. Although most existing studies that aim to model viral genotype-phenotype associations of COVID-19 use traditional statistical approaches, some leverage machine learning techniques. Studies that use machine learning by combining patient demographics and SARS-CoV-2 genome derived data such as single nucleotide polymorphisms (SNPs) or viral clades, have reported minimal impact of viral genomics on disease severity [17–19].

Associations between host factors such as age, sex, and pre-existing illnesses and severe COVID-19 have been well studied using data from electronic health records. Furthermore, numerous genome wide association studies (GWAS) revealed that host genetics are also determinants of severe disease. Large scale human genetic studies identified 13 loci involved in increasing COVID-19 susceptibility and severity [20]. However, differences in disease severity due to the interplay of viral variants combined with patient demographic and clinical profiles can complicate outcome prediction and explanatory models for COVID-19. Although millions of SARS-CoV-2 genomes have been shared publicly in global databases and enabled timely tracking of viral genetic variation and evolution, linkages between clinical data and respective SARS-CoV-2 genomes remain scarce. Given that evolutionary selection plays a critical role in determining viral fitness, genomic changes can enhance viral replication, promote host immune evasion, or decrease the effectiveness of antiviral drugs, thus influencing disease severity.

In this study, we established a dataset of COVID-19 inpatients and outpatients with combined patient and viral genomic data from the Greater Toronto Area (GTA). Electronic health data and SARS-CoV-2 whole genome sequencing were used to generate merged clinical-genomic datasets. Patient demographics, clinical factors, and SARS-CoV-2 amino acid sequences of functional genomic regions mapping to pertinent viral proteins were examined to identify associations with COVID-19 severity using machine learning (ML). Given that patient related factors such as age and pre-existing comorbidities are widely understood to influence disease severity, we aimed to investigate the added contributions of genomic determinants. We hypothesize that the combination of SARS-CoV-2 genomic data and patient factors will be predictive of COVID-19 severity. Our results show that underlying vascular and pulmonary disease and fever were the most significant clinical features associated with hospitalization. Among the genomic features that were assessed, genomic signatures found in pre-VOC variants were associated with hospitalization. Overall, patient factors were more significant in predicting hospitalization than genomic determinants.

## Methods

### Study participant recruitment and data collection

COVID-19 patients from 11 different hospital sites in the GTA were prospectively recruited by the Toronto Invasive Bacterial Disease Network (TIBDN) from March 2020 to April 2022 to the Risk of Environmental Surface and air Contamination in COVID-19 (RISC-CoV) study [21]. Patients with laboratory confirmed positive SARS-CoV-2, able to give informed consent, and who were at least 18 years of age were eligible for recruitment. All participating TIBDN hospitals were granted research ethics approval by their respective ethics boards (REB# 02-0118-U/05/0016-C). Additionally, patients from Sunnybrook Health Sciences Centre’s COVID-19 Expansion to Outpatients (COVIDEO) program [22, 23] with available specimens were retrospectively included in this study with ethics board approval (REB #5533). Patients whose specimens had a cycle threshold (Ct) value greater than 30 for the envelope (E) gene and the 5’-untranslated region (5-UTR) were excluded from further analyses. International Severe Acute Respiratory Emerging Infection Consortium (ISARIC), Randomized, Embedded, Multi-factorial, Adaptive Platform Trial for Community Acquired Pneumonia (REMAP-CAP) and TIBDN data collection tools were used for patient chart review and interviews. For COVIDEO patients, data were extracted from standardized assessment forms developed by the Infectious Diseases Division at Sunnybrook Health Sciences Centre.

### SARS-CoV-2 RNA detection

Nasopharyngeal and mid-turbinate swabs were collected and stored in universal transport media (UTM; Copan Diagnostics, Murrietta, CA) or guanidine thiocyanate-based transport media (McMaster Molecular Media, Bay Area Health Trustee Corporation, Hamilton, Ontario). All samples were processed within 24 h of receipt and the residual sample was retained for two weeks or longer. Ribonucleic acid (RNA) extraction and SARS-CoV-2 PCR detection protocols varied by clinical microbiology laboratory, all of which used validated assays and were accredited by the Institute for Quality Management in Healthcare. The majority of samples were extracted using automated liquid handlers to enable high throughput as described by Kandel et al. [24]. Samples with minimum Ct values (depending on the assay used in a given laboratory) for two SARS-CoV-2 targets were considered positive.

### Whole genome sequencing and phylogenetic analysis

Samples with Ct values less than 30 were included for whole genome sequencing, which was performed as described by Kotwa et al. [25], Nasir et al. [26], and Quick et al. [27]. In brief, cDNA was amplified using the ARTIC protocol. ARTIC V3, V4, and V4.1 primer schemes (https://github.com/artic-network/artic-ncov2019) were used in accordance with technical updates. Purified amplicons were prepared into sequencing libraries using the Nextera DNA Flex Prep Kit (Illumina, USA) following the manufacturer’s protocol. Short read paired-end (2 × 150 bp) sequencing was performed on either the MiniSeq or MiSeq instruments and a 300-cycle reagent kit (Illumina, USA). Genome assembly and subsequent analysis for short-read sequencing were performed using the SARS-CoV-2 Illumina GeNome Assembly Line (SIGNAL) standardized workflow as described by Nasir et al. [26]. Consensus genomes that met the quality thresholds of 100X depth of coverage and 75% fraction of genome coverage were assigned Pango lineages using pangolin v4.2 (PANGOLEARN 2023-03-10) [28, 29]. Variant analysis was conducted using Nextstrain Nextclade v3.0.0 [29]. The Nextstrain Augur v24.1.0 toolkit containing mafft v7.525 and IQ-TREE v2.3.0 were used for phylogenetic analyses [30].

### Clinical and genomic data pre-analytical processing and feature selection

After completing clinical chart reviews, raw chart data were cleaned to select variables common to both the inpatient and outpatient groups for further analysis. Variables with a significant amount of incomplete data were excluded. Raw data from chart review extractions from the TIBDN and COVIDEO datasets were combined, and columns were renamed to reflect consistency across clinical variables. Clinical variables included patient demographics, symptoms, and comorbidities which were one-hot encoded. Whole genome sequences were translated into amino acid sequences for each polypeptide using Nextclade [29]. Amino acid sequences of interest including the spike gene’s N-terminal domain (NTD), receptor binding domain (RBD), and S1/S2 cleavage site, open reading frame (ORF)3a, ORF8, and nucleocapsid (N) were converted into random alphanumeric characters to simplify downstream ML methods. The CoVEffect web application screens COVID-19 scientific abstracts for mutations/variants associated with epidemiological, immunological, clinical, or viral kinetic effects [31]. To identify genes of interest for downstream ML analysis, genes that appeared most frequently in the CoVEffect [31] database were selected. These genome regions are known for their importance in virus-host interactions, including their contributions to viral pathogenicity (S1/S2 cleavage site, RBD, ORF3a) [32, 33] viral entry (S1/S2 cleavage site, RBD) [34], and interaction with the host immune system (NTD, RBD, ORF3a, ORF8, N) [35–38]. The spike, ORF3a, and N genes were also selected due to the increased nucleotide diversity observed in these regions. Feature engineering and selection involved combining related variables based on clinician knowledge and the use of a correlation matrix (Supplementary Fig. 1, Additional File 1) to derive aggregate features. For example, symptoms such as anosmia and dysgeusia were aggregated into one feature.

### Statistical analysis

All categorical variables are presented as numbers by one-hot encoding. For example, for the variable cough, encoding with 1 indicates the presence of the symptom and 0 indicates its absence. Percentages and continuous variables are presented as means with standard deviations. The statistical significance between the inpatient and outpatient groups was calculated using Pearson’s chi-square test (for categorical variables) and Kruskal-Wallis rank-sum test (for continuous variables). R packages, arsenal v3.6.3 was used to summarize the set of independent variables as reported in Table 1. Entropy of each nucleotide position was calculated and a pairwise Wilcoxon rank-sum test (Mann-Whitney U test) was conducted for each pair of genes to compare entropy distributions. A Bonferroni correction was applied to the *p*-values to adjust for multiple gene comparisons of entropy values. Canonical correlation analysis (CCA) and partial least squares (PLS) techniques were used to explore cross decomposition relationships between genomic features and grouped patient symptoms.

**Table 1:** Summary of patient demographics, COVID-19 symptoms, and pre-existing medical conditions in inpatient and outpatient groups

	Inpatient (*N* = 311)	Outpatient (*N* = 306)	Total (*N* = 617)	*p* value
Sex				< 0.001
Female	126 (40.5%)	170 (55.6%)	296 (48.0%)	
Male	185 (59.5%)	136 (44.4%)	321 (52.0%)	
Age				< 0.001
Mean (SD)	66.666 (17.641)	44.886 (15.998)	55.864 (20.053)	
Range	21.000–106.000	18.000–88.000	18.000–106.000	
Fever				< 0.001
No	113 (36.3%)	183 (59.8%)	296 (48.0%)	
Yes	198 (63.7%)	123 (40.2%)	321 (52.0%)	
Chills				0.016
No	249 (80.1%)	267 (87.3%)	516 (83.6%)	
Yes	62 (19.9%)	39 (12.7%)	101 (16.4%)	
Upper respiratory tract				< 0.001
No	248 (79.7%)	183 (59.8%)	431 (69.9%)	
Yes	63 (20.3%)	123 (40.2%)	186 (30.1%)	
Cough				< 0.001
No	89 (28.6%)	167 (54.6%)	256 (41.5%)	
Yes	222 (71.4%)	139 (45.4%)	361 (58.5%)	
Shortness of breath hypoxia				< 0.001
No	84 (27.0%)	277 (90.5%)	361 (58.5%)	
Yes	227 (73.0%)	29 (9.5%)	256 (41.5%)	
Loss of smell and or taste				< 0.001
No	290 (93.2%)	244 (79.7%)	534 (86.5%)	
Yes	21 (6.8%)	62 (20.3%)	83 (13.5%)	
Productive cough				< 0.001
No	254 (81.7%)	301 (98.4%)	555 (90.0%)	
Yes	57 (18.3%)	5 (1.6%)	62 (10.0%)	
Chest pain				0.003
No	278 (89.4%)	293 (95.8%)	571 (92.5%)	
Yes	33 (10.6%)	13 (4.2%)	46 (7.5%)	
Aches				< 0.001
No	214 (68.8%)	154 (50.3%)	368 (59.6%)	
Yes	97 (31.2%)	152 (49.7%)	249 (40.4%)	
Gastrointestinal symptoms				< 0.001
No	212 (68.2%)	257 (84.0%)	469 (76.0%)	
Yes	99 (31.8%)	49 (16.0%)	148 (24.0%)	
Cardiac disease				< 0.001
No	237 (76.2%)	292 (95.4%)	529 (85.7%)	
Yes	74 (23.8%)	14 (4.6%)	88 (14.3%)	
Pulmonary disease				< 0.001
No	246 (79.1%)	303 (99.0%)	549 (89.0%)	
Yes	65 (20.9%)	3 (1.0%)	68 (11.0%)	
Asthma				0.007
No	305 (98.1%)	287 (93.8%)	592 (95.9%)	
Yes	6 (1.9%)	19 (6.2%)	25 (4.1%)	
Smoker				0.004
No	238 (76.5%)	262 (85.6%)	500 (81.0%)	
Yes	73 (23.5%)	44 (14.4%)	117 (19.0%)	
Renal disease				< 0.001
No	287 (92.3%)	300 (98.0%)	587 (95.1%)	
Yes	24 (7.7%)	6 (2.0%)	30 (4.9%)	
Liver disease				0.376
No	304 (97.7%)	302 (98.7%)	606 (98.2%)	
Yes	7 (2.3%)	4 (1.3%)	11 (1.8%)	
Neuromuscular disease				< 0.001
No	280 (90.0%)	302 (98.7%)	582 (94.3%)	
Yes	31 (10.0%)	4 (1.3%)	35 (5.7%)	
Diabetes				< 0.001
No	221 (71.1%)	278 (90.8%)	499 (80.9%)	
Yes	90 (28.9%)	28 (9.2%)	118 (19.1%)	
Vascular disease				< 0.001
No	115 (37.0%)	268 (87.6%)	383 (62.1%)	
Yes	196 (63.0%)	38 (12.4%)	234 (37.9%)	
Gastrointestinal disease				< 0.001
No	296 (95.2%)	306 (100.0%)	602 (97.6%)	
Yes	15 (4.8%)	0 (0.0%)	15 (2.4%)	
Cancer				< 0.001
No	262 (84.2%)	289 (94.4%)	551 (89.3%)	
Yes	49 (15.8%)	17 (5.6%)	66 (10.7%)	
HIV				0.321
No	310 (99.7%)	306 (100.0%)	616 (99.8%)	
Yes	1 (0.3%)	0 (0.0%)	1 (0.2%)	
Vaccinated				< 0.001
No	276 (88.7%)	239 (78.1%)	515 (83.5%)	
Yes	35 (11.3%)	67 (21.9%)	102 (16.5%)	

### Machine learning

The dataset was split whereby 70% was allocated to training with 10-fold cross validation and 30% was allocated to testing. The label for the prediction of was hospitalization, which was coded as binary: outpatient and inpatient (hospitalized). Python v3.8.18 and its packages scikit-learn 1.3.2 and XGBoost v2.0.3 were used to build supervised ML models, including logistic regression (LR), LR-least absolute shrinkage and selection operator (LR-LASSO), LR-RIDGE, decision tree (DTREE), random forest (RF), bagging (BAG), adaboost (ADB), gradient boosting machines (GBM), and XGBoost. LR-LASSO and LR-RIDGE regularization techniques were implemented to alleviate multicollinearity and improve model performance. A stacking classifier combining GBM (base model), and LR-LASSO (meta model) was evaluated for its ability to mitigate overfitting. The meta model trains on the predictions made by the GBM. The LR-LASSO as a meta model introduces regularization, which can help prevent overfitting by penalizing large coefficients and thus simplifying the model. The models’ performances were evaluated by assessing the accuracy, precision, recall, F1-score, and the area under the receiver operating characteristic curve (AUROC). Due to the consequences of undermining the false negatives, which misclassify inpatients as outpatients, recall (sensitivity) which measures the proportion of actual patients requiring hospitalization, was chosen to identify the best ML classifiers on the training dataset. We implemented the Shapley additive explanations (SHAP, v0.44.1) algorithm to present the interpretation of the model with the highest relative performance. SHAP uses cooperative game theory to calculate the marginal contribution of each feature and examines the feature influence on model prediction [39].

### Ethics approval

Informed consent was obtained and all participating TIBDN hospitals were granted research ethics approval by their respective ethics boards (REB# 02-0118-U/05/0016-C)). Use of COVIDEO patient data and specimens was approved by Sunnybrook Health Sciences Centre’s ethics board (REB# 5533).

## Results

### Baseline demographic and clinical characteristics

**Fig. 1:**
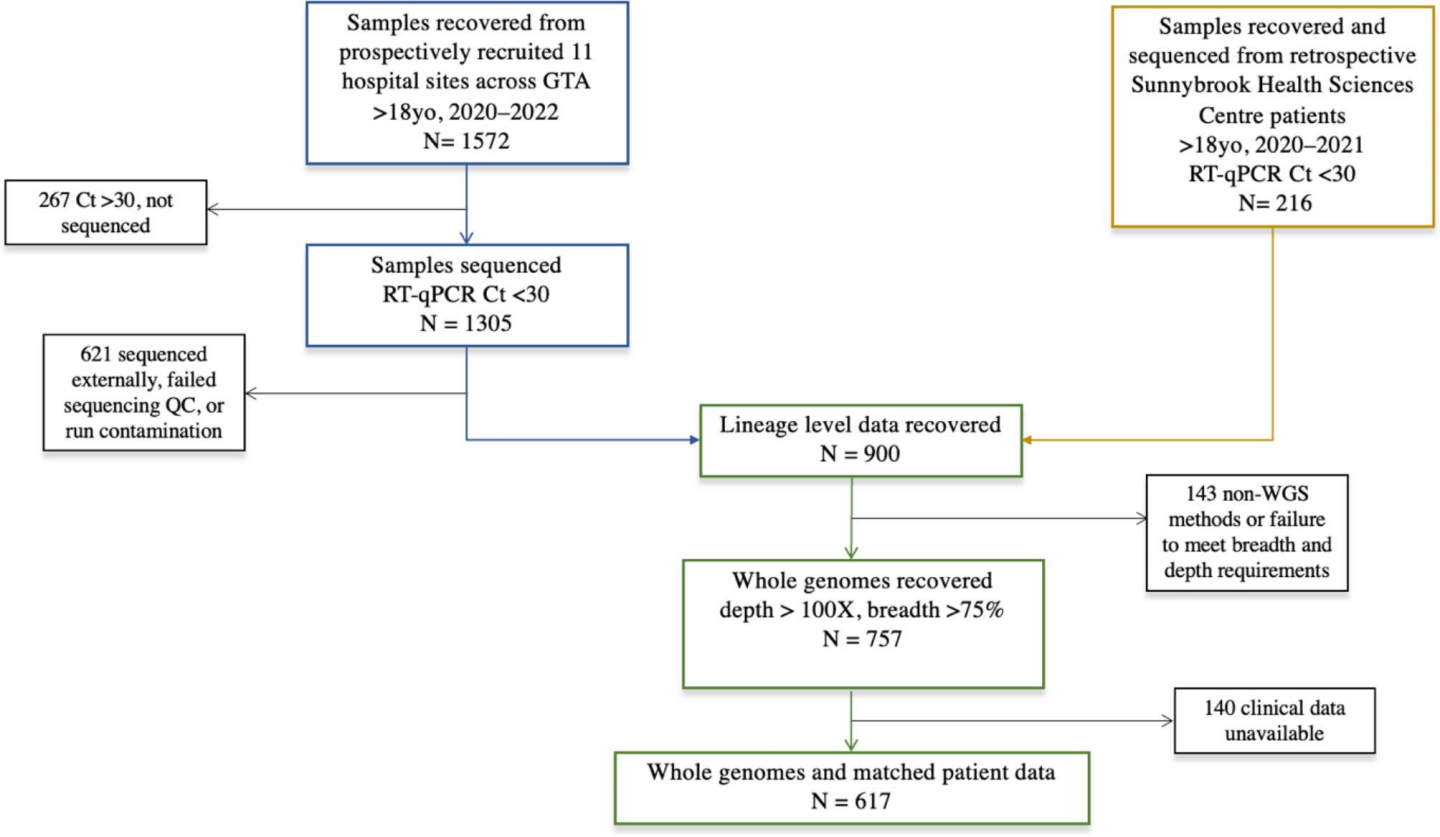
Flowchart of patient sample collection, sequencing workflows, and patient data. Laboratory confirmed SARS-CoV-2 samples from adult patients were obtained prospectively from 11 different healthcare centres in the Greater Toronto Area from March 2020 to April 2022. Separately, patients with available specimens, SARS-CoV-2 genomes, and patient data from the COVIDEO program at Sunnybrook Health Sciences Centre were added to this study retrospectively. Patients were excluded from the study if samples failed to generate quality whole genome sequences or clinical data were unavailable. A final cohort of 617 patients with matching clinical and genomic datasets was established from the two study groups

Between March 2020 and April 2022, 1572 positive COVID-19 samples were obtained from adult patients recruited from 11 different hospital sites in the GTA for the RISC-CoV study. A workflow of patient recruitment, sample processing, and data linkage is shown in Fig. 1. To increase the number of matched clinical-genomic patient datasets, patients with linked SARS-CoV-2 genomes and clinical metadata from the COVIDEO program were added to this study. Complete clinical data and viral whole genome level information were obtained from 617 patients collectively from both the RISC-CoV and COVIDEO cohorts. Key patient demographics and clinical characteristics are shown in Table 1. The mean age of the total combined clinical-genomic cohort was 55.86 years (SD ± 20.05 years) and 321 (52.0%) were male. Of the 617 patients, 311 (50.4%) were hospitalized. Inpatients were older with a mean age of 66.67 (SD ± 17.64 years) compared to outpatients with a mean age of 44.89 years (SD ± 16.00 years). Notably, pre-existing comorbidities and severe symptoms were more pronounced in the inpatient group compared to the outpatient group. The most common symptoms across the whole cohort included cough (58.5%), fever (52.0%), shortness of breath (41.5%), and myalgia (40.4%). Pre-existing comorbidities that were most predominant in the cohort included vascular disease (37.9%), diabetes (19.1%), and cardiac disease (14.3%). One hundred and two (16.5%) patients received at least 1 dose of an approved COVID-19 vaccine.

### Dynamics of circulating lineages during the study period

**Fig. 2:**
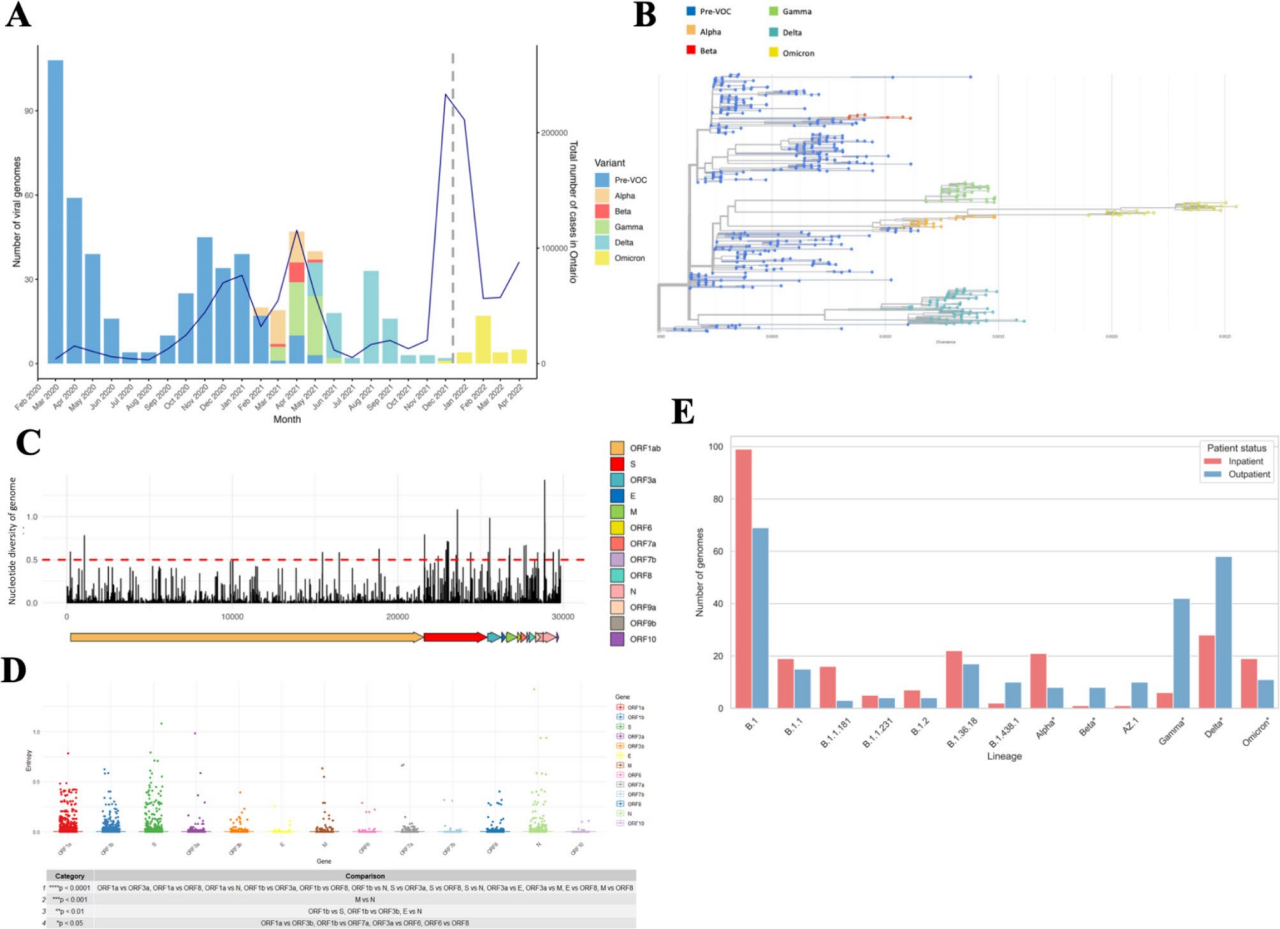
Lineage distribution and genomic diversity throughout the study period of March 2020 to April 2022. Temporal and lineage distribution of clinical-genomic matched cases. Total number of COVID-19 cases in Ontario throughout the study period are represented by the solid line. The implementation of changes to provincial testing criteria is represented by the grey dashed line (**A**). Phylogenetic tree of SARS-CoV-2 whole genomes obtained from 617 patients with consensus sequences passing 100x depth of coverage and 75% genome completeness (**B**). Nucleotide diversity per site across the whole genome (**C**). Comparison of entropy amongst SARS-CoV-2 genes. *P*-value categories highlight the statistical differences among gene regions that differ significantly in their variability based on the Wilcoxon rank-sum test (**D**). Paired comparison of number of sequences per predominant lineage by inpatient and outpatient status based on patient-genome linked 617 cases. Alpha*, Beta*, Gamma*, Delta*, and Omicron* denote sublineages as aggregate (**E**)

Throughout the study period, 69 unique Pango lineages were identified in the 617 cases with matched clinical and whole genome sequence profiles (Fig. 2a). Among the 69 lineages, 40 lineages are classified as pre-VOC variants. The VOCs Alpha, Beta, Gamma, Delta, their sub-lineages, Omicron, and its sub-lineages made up 203 (32.9%) of cases. The Alpha variant emerged in the cohort in January 2021 and dominated through the first quarter of the year. Delta peaked from June to November 2021, followed by the emergence and dominance of Omicron and its sub-variants beginning at the end of November 2021 (Fig. 2a). Nextclade [29] was used to construct a maximum likelihood phylogenetic tree from 617 consensus genomes, which were then colored by Pango lineage (Fig. 2b). The most diverse regions of the SARS-CoV-2 genomes from the 617 patients based on nucleotide diversity were located within ORF1ab, spike, ORF3a, ORF8, and N (Fig. 2c-d). The most frequent non-synonymous mutations identified in the 617 COVID-19 cases include the S: D641G mutation found in 601 (97.2%) cases, the ORF1b: P314L mutation within the non-structural protein 12 (NSP12) of the RNA-dependent RNA polymerase (RdRp) found in 592 (95.9%) cases, and the ORF3a: Q57H mutation found in 260 (42.1%) cases (Supplementary Table 1, Additional File 1). Notable VOC defining mutations such as the S: N501Y in Alpha, Beta, Gamma, and Omicron, S:E484K in Beta and Gamma, and S: P618R in Delta fall within the top 25 most predominant mutations. These mutations are located in the receptor-binding domain (S: N501Y and S: E484K) and the polybasic cleavage site (S: D614G and S: P681R). The early B.1 lineage characterized by the D614G mutation was the most predominant in both inpatients and outpatients. There were more Delta and Gamma cases in outpatients whereas the Alpha and Omicron variants were found in more inpatient cases (Fig. 2e).

### Identification of clinical and genomic factors associated with severe COVID-19 using supervised machine learning

**Fig. 3:**
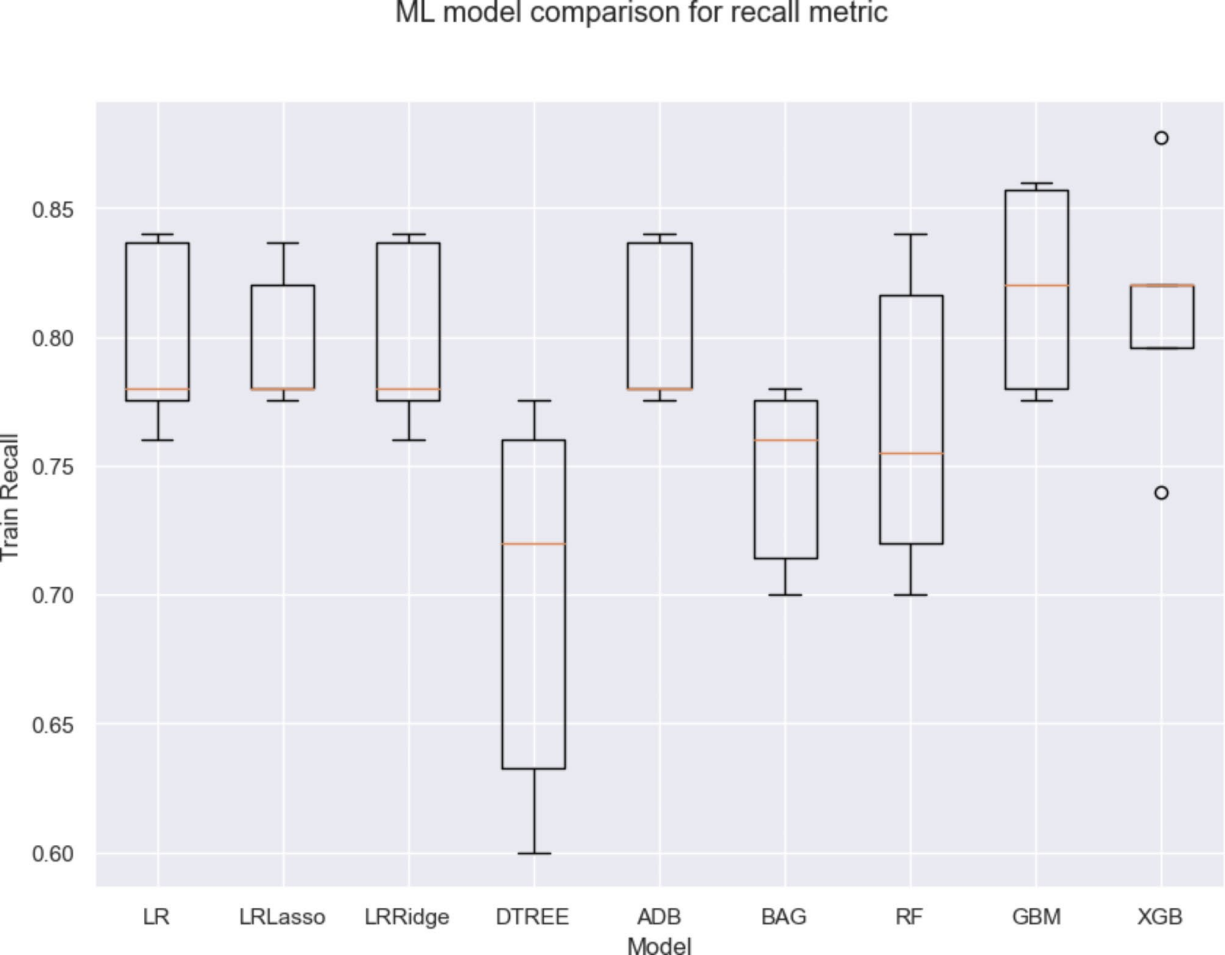
Supervised machine learning model selection and evaluation of model performance. Recall of commonly used supervised ML algorithms were compared in their ability to discriminate between inpatients and outpatients. GBM, ADB, and XGB models were among the top performers. LASSO and RIDGE regularization methods were implemented to the base LR model. LR, logistic regression; BAG, bootstrap aggregating; RF, random forest; GBM, gradient boosting machines; ADB, adaboost; XGB, XGBoost; DTREE, decision tree; LASSO, least absolute shrinkage and selection operator

**Fig. 4:**
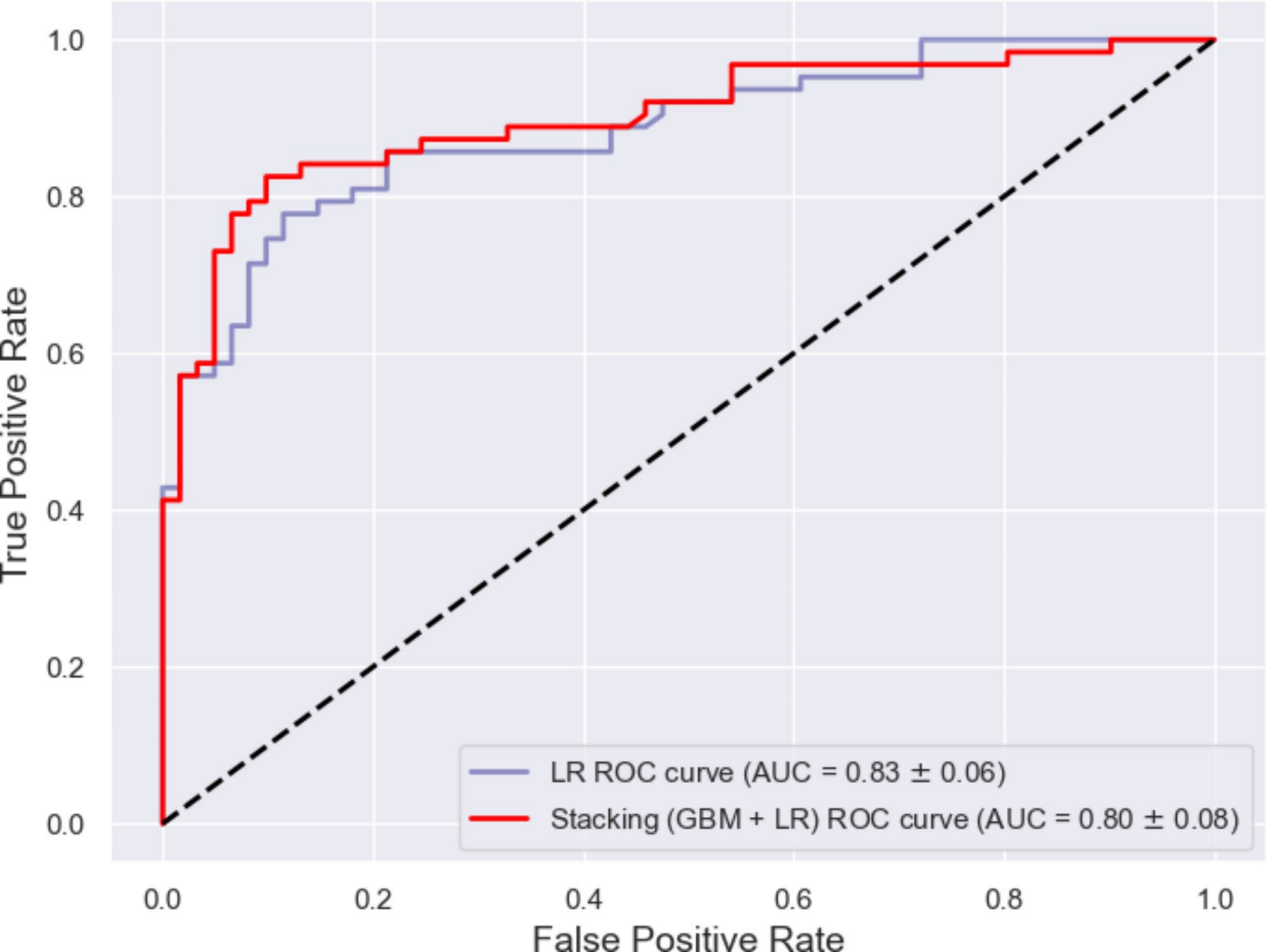
Performance evaluation of test dataset LR and Stacking (GBM + LR-LASSO) models. Receiver operating characteristic (ROC) curve for the prediction of hospitalization

Supervised ML was used to train predictive models for hospitalization to determine the importance of clinical and viral genomic features. Amino acid sequences of SARS-CoV-2 proteins with functional relevance to viral fitness, pathogenicity, and immune escape were also directly tested for statistical association with hospitalization status along with patient demographics, underlying comorbidities, and presenting symptoms. We used the CoVEffect^26^ database to refine our selection of proteins to those most frequently associated with functional effects in the literature. We focused on the highly mutated spike protein and immune modulating proteins ORF3a, ORF8, and N. Results based on the spike protein’s NTD, S1/S2 cleavage site, and RBD are presented further. Supplementary Fig. 2, Additional File 1 summarizes the outputs from the ORF3a, ORF8, and N based models. Nine ML algorithms were deployed and evaluated on the basis of their ability to predict patient hospitalization status using clinical and SARS-CoV-2 genome-derived data (Fig. 3). Due to the consequences of undermining the model’s false negative rate which would misclassify inpatients as outpatients, assessment of the recall metric was prioritized. Overall, the LR model consistently outperformed other classifiers (LR-LASSO, GBM, XGB and stacking) across the evaluation metrics (Table 2), suggesting that it generalized well without overfitting. However, the GBM and XGB models failed to achieve comparable consistency across evaluation metrics, indicating potential issues with generalization (or overfitting). We further explored a stacking classifier that combined GBM (as the base model) and LR-LASSO (as the meta model) to improve both training and test performance, aiming to mitigate the overfitting issues observed in these individual models. However, this stacking model did not outperform the LR model but still performed well, particularly in terms of test recall (0.8115 ± 0.1024), which is comparable to the top performing LR model. This suggests that the stacking model provided complementary strengths that allowed efficient identification of true positives while maintaining reasonable precision. However, the slightly lower accuracy (0.80 ± 0.08) on the test set compared to LR, suggests slightly less robustness in generalizing unseen data. This exploration demonstrates that the stacking approach remains valuable for leveraging the diverse capabilities of both GBM and LR-LASSO to balance model performance. Figure 4 presents the test AUROC curves of the LR model achieving the highest area under the curve (AUC) of 0.83 ± 0.06, followed by the stacking model with an AUC of 0.80 ± 0.08. The AUROC curves of the other base models are presented in Supplementary Fig. 3, Additional File 1.

**Table 2:** Training and test set accuracy, recall, precision, and F1 score of base and stacked models

Model	Training Accuracy	Test Accuracy	Training Precision	Test Precision	Training Recall	Test Recall	Training F1	Test F1
LR	0.83 ± 0.05	0.83 ± 0.06	0.85 ± 0.05	0.86 ± 0.09	0.81 ± 0.06	0.81 ± 0.10	0.83 ± 0.05	0.83 ± 0.07
Stacking (GBM + LR_LASSO)	0.83 ± 0.05	0.80 ± 0.06	0.85 ± 0.05	0.82 ± 0.11	0.81 ± 0.05	0.81 ± 0.10	0.83 ± 0.05	0.81 ± 0.06
GBM	0.83 ± 0.04	0.78 ± 0.05	0.84 ± 0.06	0.80 ± 0.10	0.82 ± 0.06	0.80 ± 0.10	0.83 ± 0.05	0.79 ± 0.05
LR_LASSO	0.82 ± 0.04	0.83 ± 0.05	0.84 ± 0.04	0.89 ± 0.07	0.79 ± 0.05	0.78 ± 0.11	0.81 ± 0.04	0.82 ± 0.05
XGBoost	0.83 ± 0.05	0.80 ± 0.09	0.85 ± 0.05	0.84 ± 0.13	0.80 ± 0.07	0.78 ± 0.03	0.82 ± 0.05	0.80 ± 0.07

**Fig. 5:**
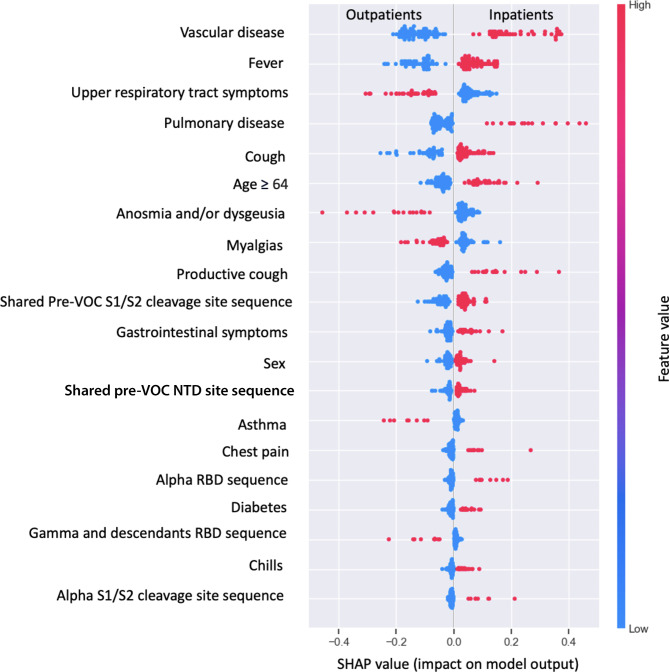
Clinical, demographic, and SARS-CoV-2 genomic features ranked in descending order of importance in predicting hospitalization. Summary plot of feature ranking and positive and negative influence in predicting hospitalization based on SHAP values derived from the optimal LR model. Underlying vascular illness, fever, upper respiratory tract symptoms, underlying pulmonary disease, and cough have the greatest impact on model prediction. A high feature and positive SHAP value as observed in vascular disease, fever, pulmonary disease, and cough have a positive impact on model prediction, suggesting the prediction of inpatients. Conversely, high feature values with negative SHAP values indicate a negative impact on model prediction, which can be interpreted as predicting outpatients. Thus, upper respiratory tract symptoms, myalgias, and anosmia and/or dysgeusia have a negative impact on predicting hospitalization, which suggests association with outpatients. “Shared” denotes lineages that have 100% amino acid sequence similarity across multiple lineages. The pre-VOC S1/S2 cleavage site sequence group denotes 30 lineages that are descendants of B.1 which circulated prior to VOC emergence that share the same amino acid sequence at the S1/S2 cleavage site

To further investigate the importance of model features in predicting hospitalization based on the optimal model, SHAP was used to interpret the results of the top performing LR model. The top features that ranked the highest in their ability to discriminate between inpatients and outpatients were underlying vascular or pulmonary disease, fever, upper respiratory tract symptoms, and cough. (Fig. 5). SHAP was also applied to the other models (Stacking and XGBoost) whereby the same features were ranked at the top consistently across models (Supplementary Fig. 4, Additional File 1). The SHAP summary plot describes whether features have a negative or positive influence on prediction and the magnitude of feature effect. Furthermore, red indicates high feature values, whereas blue indicates low feature values. High feature and positive SHAP values, as observed in underlying vascular and pulmonary disease and fever indicate high contribution to classifying/predicting inpatients (Fig. 5). In contrast, high feature values and negative SHAP values, as observed in upper respiratory tract symptoms (rhinorrhea and sore throat), myalgias and anosmia and/or dysgeusia contribute to underestimating association with hospitalization and thus indicate association with outpatients (Fig. 5). Interestingly, amino acid sequences associated with predicting hospitalization were pre-VOC lineages that share identical amino acid sequences within the S1/S2 cleavage site. There are 30 lineages that are descendants of B.1 that share the same sequence of amino acids at the spike S1/S2 cleavage site (Supplementary Table 2, Additional File 1). CCA and PLS techniques were used to investigate potential relationships between genomic features and patient symptoms. The CCA resulted in a canonical correlation coefficient of 0.38, suggesting weak to moderate correlation between genomic features and grouped symptoms. Based on the PLS analysis, the highest loadings were observed in genomic features associated with the Delta lineage suggesting significant contributions to the first component (Supplementary Fig. 5, Additional File 1).

## Discussion

This study combines demographic and clinical patient data with SARS-CoV-2 genomes to predict hospitalization using supervised ML and then investigates relative feature importance. Unsurprisingly, patient features including underlying vascular and pulmonary disease and fever more strongly predicted hospitalization than did viral genomic features. Patient features including upper respiratory tract symptoms and anosmia or dysgeusia were associated with outpatient status. Although we found that overall genomic features were less impactful in predicting hospitalization, the S1/S2 cleavage site sequences from pre-VOC variants were associated with hospitalization.

COVID-19 disease presentation is multifactorial and varies widely among individuals due to the interplay of host, viral, and environmental factors [4]. Age and comorbidities such as hypertension, cardiovascular disease, and diabetes were established as risk factors for severe COVID-19 early in the pandemic [3]. Systematic review and meta-analysis revealed that comorbidities, including hypertension and chronic obstructive pulmonary disease, and cough, were predictive of severe disease [4]. Similarly, we found that pre-existing vascular and pulmonary diseases and cough are predictive of hospitalization, which is consistent with existing studies [1, 3, 4, 6]. We found that upper respiratory tract symptoms such as rhinorrhea, sore throat, anosmia, and dysgeusia were associated with outpatient status and by inference, less severe disease. Similarly, studies have shown that olfactory dysfunction is more prevalent in mild-to-moderate COVID-19 patients and that 68.4% of patients are younger than those without these symptoms [40]. A systematic review and meta-analysis found that upper respiratory tract symptoms such as sore throat were experienced significantly more frequently by non-hospitalized patients than hospitalized patients, which aligns with our findings [29, 41].

Various studies have employed ML to predict SARS-CoV-2 disease severity using mutational features. Mutational signatures within the spike protein V1176F (heptapeptide repeat sequence 2) and S477N (RBD) which co-occur with D614G (S1/S2 cleavage site) were associated with the prediction of severe COVID-19 with the use of ML [42, 43]. Such SNPs found in the RBD (S477N) and S1/S2 cleavage site (D614G) are known to impact viral entry and pathogenesis [32, 34]. This is consistent with our findings which suggest that the amino acid sequence shared among pre-VOC variants is associated with hospitalization. In the present study, rather than using SNPs as genomic features in our ML models, we used the amino acid sequences of these key genome regions as inputs into our models. This mitigates noise from overlapping mutations across the SARS-CoV-2 genome shared among multiple differing lineages.

A growing number of studies have also evaluated the ability of SARS-CoV-2 mutations to predict the severity of illness. Fewer studies use ML with linked SARS-CoV-2 genomic and clinical datasets. Foxely-Marrable et al. combined patient demographics and comorbidities with SARS-CoV-2 synonymous and nonsynonymous mutations to identify risk factors in predicting disease severity using ML [17]. Consistent with our viral genomic findings, Foxley-Marrable et al. suggested that specific SARS-CoV-2 mutations are unlikely to significantly impact disease severity. However patient symptoms were not included in their dataset. Furthermore, many studies that include SARS-CoV-2 genomic data, presents them as entire lineages or individual SNPs. Our approach directly uses amino acid sequences, which in turn represent constellations of mutations present in each SARS-CoV-2 protein. Proteins with known involvement in vital processes such as viral entry, pathogenicity, and host immune escape were included in our ML models. We used the CoVEffect [31] database, which employs natural language processing to mine COVID-19 scientific abstracts for mutations/variants associated with epidemiological, immunological, clinical, or viral kinetic effects. In addition to the spike protein, we investigated proteins with the most mutations associated with functional effects, as indicated by the CoVEffect database, which included N, ORF3a, and ORF8. The N protein has been implicated in increased viral infectivity, replication, and pathogenesis as observed in variants possessing R204K/G204R mutations including Alpha (B.1.1.7) [44]. Interestingly, in our models, both the spike RBD and the N protein sequence of Alpha indicated association with disease severity (Fig. 5 and Supplementary Fig. 2, Additional File 1). The N protein is also a known immune system antagonist involved in impeding interferon signaling [45]. ORF3a is an inducer of innate immunity and proinflammatory responses that can lead to cytokine dysregulation, particularly under hypoxic conditions [33]. ORF3a also plays a role in tissue damage by inducing cell death [46]. ORF8 has been implicated in the downregulation of MHC class I expression to evade T-cell recognition [38].

There are several limitations in this study. Although 1,788 patient samples were initially obtained, only 617 were included due to the availability of matched clinical and high-quality genomic data.

The final cohort which was specifically recruited from the Greater Toronto Area may limit generalizability to larger populations or different geographic areas. Nonetheless, our findings highlight that patient factors such as underlying comorbidities are significant predictors of severe disease. This is consistent with existing studies. However, the magnitude of specific genomic contributions in predicting disease severity may not be appropriately applicable to broader populations given our constrained sample size and geographic restrictions. A significant number of samples were excluded due to high Ct values, failed sequencing runs, sequencing performed at external laboratories, run contamination, partial genome sequencing, failure to meet depth and breadth of coverage cut-offs, and missing clinical data. As a result, this limited statistical power may introduce sampling bias and thereby skew generalizability. Most patients that were excluded from the study were inpatients who may have presented after symptom onset due to progressive illness, thus viral shedding may have been insufficient to yield high quality viral sequences. A very small subset of patients presented to testing centers as outpatients during early symptom onset but were later admitted to hospital due to illness progression. These patients were flagged during clinical chart review and coded as inpatients. Time to hospital admission and length of hospital stay were not included in the models as these variables were not available for outpatients.

High testing volumes and limited resources enabling sample retention during heightened pandemic waves resulted in a significant attrition of patients. We acknowledge that the cases represented in our study may not have accurately reflected the ratio of outpatient vs. hospitalized cases during pandemic waves due to changes in testing criteria, thus presenting collider biases. For example, there are more inpatient than outpatient cases of Omicron (Fig. 2e), which is a result of changes to provincial testing regulations that only permit PCR testing for high risk individuals. In the province of Ontario, this change was made effective as of December 31, 2021 during Omicron’s first wave. It is widely understood that Omicron is a less severe SARS-CoV-2 variant and there was increased vaccine uptake and outpatient Paxlovid use during this pandemic wave [47]. This form of sampling bias can distort potential associations between hospitalization and patient and/or genomic factors. We recognize that most patients infected with pre-VOC lineages were not vaccinated and therefore the impact vaccines have on reducing severe disease in these lineages are uncertain. Only 16.5% of patients were vaccinated (Table 1), which was accounted for in our modeling. Given that we did not longitudinally follow our cohort over time, we do not have accurate information on all patients’ vaccination history beyond the first dose. This may limit the impact of our analysis as we were unable to comprehensively include complete vaccination history or evaluate the effects of breakthrough infections following vaccination or reinfection. Since a small fraction of our cohort was vaccinated, we speculate that vaccination may have limited impacts on our models as vaccines were not yet available when early pandemic variants were in circulation. Additionally, our analysis does not include viral load data, which are known to impact disease severity.

It is important to note that the study period only captures SARS-CoV-2 diversity from March 2020 to April 2022, thereby potentially limiting the applicability of our findings to currently circulating SAR-CoV-2 variants. Inclusion of more ancestral genotypes of SARS-CoV-2 underscores the severity of infection prior to viral adaptation in human populations. This framework of combining patient factors with genomic signatures may be less relevant to today’s contemporary Omicron sublineages due to their narrow diversity and incremental evolutionary changes compared to the broad lineage diversity observed pre-Omicron. Nonetheless, mobilizing machine learning frameworks such as ours which combine patient factors with viral genomic data would be effective in the case of a new viral pandemic to predict severe disease particularly where linked genomic and clinical data become available in a timely manner. This will support early prioritization and prediction of severe cases while classifying and phenotyping emerging viral variants. Another limitation is that the dependent variable in our models is classified as binary, where patients fall into either inpatients or outpatients to indicate disease severity. Notably, some patients were admitted to hospital due to increased risks of severe illness such as old age or frailty despite not experiencing severe COVID-19. The use of an ordinal scale to classify disease severity such as the WHO clinical progression scale [48] would address this limitation as patients who are hospitalized without intervention would be assigned a lower score than those receiving treatment. Due to the limited number of cases in our final clinical-genomic matched cohort, which reflects an additional limitation, an ordinal scale was not applied to our models. Given the notable difference in age and sex of our inpatient and outpatient groups, we simulated a 1:1 matched cohort using propensity score matching to alleviate these imbalances (Supplementary Fig. 6, Additional File 1). Although age and sex became balanced, the sample size reduced significantly from 617 to 332 and downstream ML analyses performed poorly. However, similar SHAP patterns in which the prediction of hospitalization was driven by underlying vascular disease, cough and fever were consistent with the unmatched dataset. Furthermore, the CoVEffect tool used to select viral genomic features does not capture the whole SARS-CoV-2 genome. As a result, rare or individual mutations were not considered due to the lack of statistical power within our dataset. We acknowledge that including genes that appeared most frequently in the literature may bias our findings as we may miss other relevant genomic features. Although correlating viral genomic attributes with hospitalization is a focus of our study, the addition of host genomics would enhance our understanding of relationships at the virus-host interface. GWAS was not within the scope of this project but could be an avenue to explore in future work.

## Conclusions

The findings of this study provide insight into understanding COVID-19 disease severity based on combined patient related factors including demographic and clinical features, along with SARS-CoV-2 genomic data. Rather than assessing SARS-CoV-2 lineages, SNPs or a combination of mutations throughout the whole genome, we explored the relationship of genome regions with known functional relevance to virulence, antigenicity, and interaction with the host immune system as identified in the growing body of literature. With our supervised ML approach, we found that patient related factors have greater discriminatory power in distinguishing inpatients and outpatients than SARS-CoV-2 genomic data. These data suggest that SARS-CoV-2 genomic factors alone are not predictive of disease severity. However, it cannot be ruled out that viral determinants are not associated with disease severity [6, 11–13]. Nonetheless, linking patient data with SARS-CoV-2 genomes provides an enriched perspective in understanding the roles of clinical and viral factors on disease.

## Electronic supplementary material

Below is the link to the electronic supplementary material.

Supplementary Material 1

Supplementary Material 2

## Data Availability

SARS-CoV-2 genomes analyzed in this study can be accessed from the Global Initiative on Sharing All Influenza Data (GISAID) available at https://www.gisaid.org. See Additional File 2 for the accession numbers of the consensus genomes deposited to GISAID. De-identified processed and analyzed data can be obtained upon reasonable request from the corresponding author if approval from ethics boards of all centers involved is granted.

## Abbreviations

ADB: Adaboost
AUROC: Area under the receiver operating characteristic
BAG: Bagging
CCA: Canonical correlation analysis
COVID-19: Coronavirus disease 2019
COVIDEO: COVID-19 Expansion to Outpatients
Ct: Cycle threshold
DTREE: Decision tree
GBM: Gradient boosting machines
GTA: Greater Toronto Area
GWAS: Genome wide association studies
ISARIC: COVID-19 Expansion to Outpatients
LR-LASSO: LR-least absolute shrinkage and selection operator
LR: Logistic regression
MHC: Major histocompatibility complex
ML: Machine learning
N: Nucleocapsid
NTD: N-terminal domain
ORF: Open reading frame
PLS: Partial least squares
RBD: Receptor binding domain
REMAP-CAP: Randomized, Embedded, Multi-factorial, Adaptive Platform Trial for Community Acquired Pneumonia
RF: Random forest
RISC-CoV: RIsk of Environmental Surface and air Contamination in COVID-19
SARS-CoV-2: Severe acute respiratory syndrome coronavirus-2
SHAP: Shapley additive explanations
SIGNAL: SARS-CoV-2 Illumina GeNome Assembly Line
SNPs: Single nucleotide polymorphisms
TIBDN: Toronto Invasive Bacterial Disease Network
VOCs: Variants of concern
WHO: World Health Organization
