# Correction: Identification of patient demographic, clinical, and SARS-CoV-2 genomic factors associated with severe COVID-19 using supervised machine learning: a retrospective multicenter study

**DOI:** 10.1186/s12879-025-11828-z

**Published:** 2025-10-16

**Authors:** Kuganya Nirmalarajah, Patryk Aftanas, Shiva Barati, Emily Chien, Gloria Crowl, Amna Faheem, Lubna Farooqi, Alainna J. Jamal, Saman Khan, Jonathon D. Kotwa, Angel X. Li, Mohammad Mozafarihashjin, Jalees A. Nasir, Altynay Shigayeva, Winfield Yim, Lily Yip, Xi Zoe Zhong, Kevin Katz, Robert Kozak, Andrew G. McArthur, Nick Daneman, Finlay Maguire, Allison J. McGeer, Venkata R. Duvvuri, Samira Mubareka

**Affiliations:** 1 Sunnybrook Research Institute, Toronto, ON Canada; 2 Shared Hospital Laboratory, Toronto, ON Canada; 3 Sinai Health System, Toronto, ON Canada; 4 North York General Hospital, Toronto, ON Canada; 5 Faculty of Computer Science, Dalhousie University, Halifax, NS Canada; 6 Department of Community Health & Epidemiology, Faculty of Medicine, Dalhousie University, Halifax, NS Canada; 7 Michael G. DeGroote Institute for Infectious Disease Research, McMaster University, Hamilton, ON Canada; 8 Department of Biochemistry and Biomedical Sciences, McMaster University, Hamilton, ON Canada; 9 Public Health Ontario, 661 University Avenue, Toronto, ON Canada; 10 Department of Laboratory Medicine & Pathobiology, University of Toronto, Toronto, ON Canada; 11 Laboratory for Industrial and Applied Mathematics, Department of Mathematics and Statistics, York University, Toronto, ON Canada

**Correction: Nirmalarajah et al. BMC Infectious Diseases 25, 132 (2025)**

**https://doi.org/10.1186/s12879-025–10450-3**

Following publication of the original article [1], the authors have flagged a correction for Fig. 5, where the label “Gamma and descendants RBD sequence” appeared twice. The first occurrence was changed to “Shared pre-VOC NTD site sequence”.

The original article has been corrected.
