# Supplementary material for: Identification of patient demographic, clinical, and SARS-CoV-2 genomic factors associated with severe COVID-19 using supervised machine learning: a retrospective multicenter study
Source: BMC Infect Dis. 2025 Jan 28;25:132. doi: 10.1186/s12879-025-10450-3 (PMC11773898; doi:10.1186/s12879-025-10450-3)
Supplement: Supplementary file 1 — Supplementary Material 1 [file 12879_2025_10450_MOESM1_ESM.pdf]

Supplementary

Table of Contents

Supplementary Table 1: Top 25 non-synonymous mutations……………………..………………1

Supplementary Table 2: Genomic features from machine learning models…………………….2-4

Supplementary figure 1: Correlation matrix of clinical variables………………………………...5

Supplementary Figure 2: SHAP analysis of Nucleocapsid, ORF3a, and ORF8 proteins………6-9

Supplementary figure 3: ROC curve for GBM, LR LASSO, and XGB models………………..10

Supplementary Figure 4: SHAP analysis using LR and XGBoost models…………………..11-13

Supplementary Figure 5: PLS analysis……………………………………………………..14

Supplementary Figure 6: Propensity score matching to control for age and sex….……..…..15-16

Supplementary Table 1: Top 25 non-synonymous mutations identified in inpatient and outpatient groups from the 617 cases.

Outpatients		Inpatients	
**Mutation**	**Count**	**Mutation**	**Count**
**S:D614G**	300	S:D614G	301
**ORF1ab:P314L**	292	ORF1ab:P314L	300
**ORF3a:Q57H**	119	ORF3a:Q57H	141
**N:R203K**	106	N:R203K	111
**N:G204R**	104	N:G204R	110
**M:I82T**	69	ORF1ab:T265I	77
**S:T478K**	67	S:N501Y	46
**ORF1ab:T265I**	66	S:T478K	46
**S:N501Y**	63	N:S194L	45
**S:D950N**	58	S:P681H	41
**N:D377Y**	58	ORF1ab:T3255I	31
**ORF1ab:G662S**	58	M:I82T	29
**ORF1ab:P1000L**	58	ORF3a:S26L	29
**S:P681R**	58	ORF9b:A29T	29
**N:R203M**	58	S:P681R	29
**N:D63G**	58	N:R32H	29
**ORF9b:T60A**	58	S:D950N	28
**ORF3a:S26L**	58	N:D63G	28
**S:T19R**	58	ORF9b:T60A	28
**ORF7a:T120I**	57	N:D377Y	28
**ORF7a:V82A**	57	ORF1ab:G662S	28
**S:R158G**	56	ORF1ab:P1000L	28
**S:E484K**	56	S:T19R	28
**S:L452R**	56	N:R203M	27
**S:H655Y**	54	ORF7a:T120I	27

Supplementary Table 2: Lineages and amino acid sequences of genomic features described in the machine learning analysis.

Sequence description	Alphanumeric Code	Lineages	Amino Acid Sequence
**Shared pre-VOC S1/S2 cleavage site sequence**	cleavage_Y9O9HG	AE.8, B.1, B.1.1, B.1.1.1, B.1.1.121, B.1.1.157, B.1.1.176, B.1.1.181, B.1.1.216, B.1.1.231, B.1.1.33, B.1.1.413, B.1.1.417, B.1.1.434, B.1.111, B.1.128, B.1.160, B.1.2, B.1.265, B.1.346, B.1.349, B.1.350, B.1.351, B.1.36, B.1.36.18, B.1.36.31, B.1.36.38, B.1.36.8, B.1.427, B.1.523	NFNGLTGTGVLTESNKKFLPFQQFGRDIADTTDAVRDPQTLEILDITPCSFGGVSVITPGTNTSNQVAVLYQGVNCTEVPVAIHADQLTPTWRVYSTGSNVFQTRAGCLIGAEHVNNSYECDIPIGAGICASYQTQTNSPRRAR
**Shared pre-VOC NTD site sequence**	ntd_7GVOUX	A.1, A.3, AE.8, B, B.1, B.1.1, B.1.1.1, B.1.1.121, B.1.1.157, B.1.1.181, B.1.1.216, B.1.1.231, B.1.1.33, B.1.1.434, B.1.111, B.1.128, B.1.160, B.1.2, B.1.243, B.1.349, B.1.350, B.1.36, B.1.36.38, B.1.438.1, B.1.523, B.3, C.36	TRTQLPPAYTNSFTRGVYYPDKVFRSSVLHSTQDLFLPFFSNVTWFHAIHVSGTNGTKRFDNPVLPFNDGVYFASTEKSNIIRGWIFGTTLDSKTQSLLIVNNATNVVIKVCEFQFCNDPFLGVYYHKNNKSWMESEFRVYSSANNCTFEYVSQPFLMDLEGKQGNFKNLREFVFKNIDGYFKIYSKHTPINLVRDLPQGFSALEPLVDLPIGINITRFQTLLALHRSYLTPGDSSSGWTAGAAAYYVGYLQPRTFLLKYNENGTIT
**Gamma and descendants RBD sequence**	rbd_MQDRG1	P.1, P.1.14, P.1.17	GEVFNATRFASVYAWNRKRISNCVADYSVLYNSASFSTFKCYGVSPTKLNDLCFTNVYADSFVIRGDEVRQIAPGQTGTIADYNYKLPDDFTGCVIAWNSNNLDSKVGGNYNYLYRLFRKSNLKPFERDISTEIYQAGSTPCNGVKGFNCYFPLQSYGFQPTYGVGYQPYRVVVLSFELLHAPATV
**Shared pre-VOC and Delta sublineages RBD sequence**	rbd_9SX68V	AY.127, AY.74, AZ.1, B.1, B.1.1, B.1.1.157, B.1.1.231, B.1.1.417, B.1.265, B.1.349, B.1.36, B.1.36.18, B.1.36.8, B.1.438.1	GEVFNATRFASVYAWNRKRISNCVADYSVLYNSASFSTFKCYGVSPTKLNDLCFTNVYADSFVIRGDEVRQIAPGQTGKIADYNYKLPDDFTGCVIAWNSXXXXXXXXXXXXXXXXXXXXXXXXXXXXXXXXXXXXXXXXXXXXXXXXXXXXXXXXXXXXXXXXXXXXXXXXXXXXXXXXXX
**Alpha RBD sequence**	rbd_CVNBH6	B.1.1.7	GEVFNATRFASVYAWNRKRISNCVADYSVLYNSASFSTFKCYGVSPTKLNDLCFTNVYADSFVIRGDEVRQIAPGQTGKIADYNYKLPDDFTGCVIAWNSNNLDSKVGGNYNYLYRLFRKSNLKPFERDISTEIYQAGSTPCNGVEGFNCYFPLQSYGFQPTYGVGYQPYRVVVLSFELLHAPATV
**Alpha S1/S2 cleavage site sequence**	cleavage_17CW4X	B.1.1.7	NFNGLTGTGVLTESNKKFLPFQQFGRDIDDTTDAVRDPQTLEILDITPCSFGGVSVITPGTNTSNQVAVLYQGVNCTEVPVAIHADQLTPTWRVYSTGSNVFQTRAGCLIGAEHVNNSYECDIPIGAGICASYQTQTNSHRRAR

**Gamma and descendants S1/S2 cleavage site sequence**	cleavage_7F7LK0	P.1, P.1.14, P.1.17	NFNGLTGTGVLTESNKKFLPFQQFGRDIADTTDA VRDPQTLEILDITPCSFGGVSVITPGTNTSNQVAV LYQGVNCTEVPVAIHADQLTPTWRVYSTGSNVF QTRAGCLIGAEYVNNSYECDIPIGAGICASYQTQ TNSPRRAR
**B.1.36.8**	N_P7FMF7	B.1.36.8	MSDNGPQNQRNAPRITFGGPSDSTGSNQNGERS GARSKQRRPQGLPNNTASWFTALTQHGKEDLKF PRGQGVPINTNSSPDDQIGYYRRATRRIRGGDGK MKDLSPRWYFYYLGTGPEAGLPYGANKDGIIW VATEGALNTPKDHIGTRNPANNAAIVLQLPQGT TLPKGFYAEGSRGGSQASSRSSSRSRNSLRNSTP GSSRGTSPARMAGNGGDAALALLLLDRLNQLES KMSGKGQQQQGQTVTKKSAAEASKKPRQKRTA TKAYNVTQAFGRRGPEQTQGNFGDQELIRQGTD YKHWPQIAQFAPSASAFFGMSRIGMEVTPSGTW LTYTGAIKLDDKDPNFKDQVILLNKHIDAYKIFP PTEPKKDKKKKADETQALPQRQKKQQTVTLLPA ADLDDFSKQLQQSMSSADSTQA*
**Alpha**	N_CCEBG2	B.1.1.7	MSLNGPQNQRNAPRITFGGPSDSTGSNQNGERS GARSKQRRPQGLPNNTASWFTALTQHGKEDLKF PRGQGVPINTNSSPDDQIGYYRRATRRIRGGDGK MKDLSPRWYFYYLGTGPEAGLPYGANKDGIIW VATEGALNTPKDHIGTRNPANNAAIVLQLPQGT TLPKGFYAEGSRGGSQASSRSSSRSRNSSRNSTP GSSKRTSPARMAGNGGDAALALLLLDRLNQLES KMFGKGQQQQGQTVTKKSAAEASKKPRQKRTA TKAYNVTQAFGRRGPEQTQGNFGDQELIRQGTD YKHWPQIAQFAPSASAFFGMSRIGMEVTPSGTW LTYTGAIKLDDKDPNFKDQVILLNKHIDAYKTFP PTEPKKDKKKKADETQALPQRQKKQQTVTLLPA ADLDDFSKQLQQSMSSADSTQA*
**B.1.438.1**	N_OOR4GE	B.1.438.1	MSDNGPQNQRNAPRITFGGPSDSTGSNQNGERS GARSKQRRPQGLPNNTASWFTALTQHGKEDLKF PRGQGVPINTNSSPDDQIGYYRRATRRIRGGDGK MKDLSPRWYFYYLGTGPEAGLPYGANKDGIIW VATEGALNTPKDHIGTRNPANNAAIVLQLPQGT TLPKGFYAEGSRGGSQASSRSSSRSRNSSRNSTP GSSRGTSPARMAGNGGDAALALLLLDRLNQLES KISGKGQQQQGQTVTKKSAAEASKKPRQKRTAT KAYNVTQAFGRRGPEQTQGNFGDQELIRQGTDY KHWPQIAQFAPSASAFFGMSRIGMEVTPSGTWL TYTGAIKLDDKDPNFKDQVILLNKHIDAYKTFPP

			TEPKKDKKKKADETQALPQRQKKQQTVTLLPA<br>ADLYDFSKQLQQSMSSADSTQA*
**Shared pre-VOC ORF3a lineages**	orf3a_GR6CKB	A.1, A.23.1, A.3, AE.8, AZ.1, B.1, B.1.1, B.1.1.1, B.1.1.121, B.1.1.157, B.1.1.181, B.1.1.33, B.1.1.413, B.1.1.417, B.1.1.7, B.1.128, B.1.243, B.3, BA.1, BA.1.1, BA.1.1.10, BA.2, C.36	MDLFMRIFTIGTVTLKQGEIKDATPSDFVRATATI<br>PIQASLPFGWLIVGVALLAVFQSASKIITLKKRW<br>QLALSKGVHFVCNLLLLFVTVYSHLLLVAAGLE<br>APFLYLYALVYFLQSINFVRIIMRLWLCWKCRSK<br>NPLLYDANYFLCWHTNCYDYCIPYNSVTSSIVIT<br>SGDGTTSPISEHDYQIGGYTEKWESGVKDCVVL<br>HSYFTSDYYQLYSTQLSTDTGVEHVTFFIYNKIV<br>DEPEEHVQIHTIDGSSGVVNPVMEPIYDEPTTTTS<br>VPL*
**Shared pre-VOC ORF3a lineages**	orf3a_0EITLO	B.1, B.1.111, B.1.160, B.1.346, B.1.349, B.1.350, B.1.36, B.1.36.18, B.1.36.38, B.1.427, B.1.523	MDLFMRIFTIGTVTLKQGEIKDATPSDFVRATATI<br>PIQASLPFGWLIVGVALLAVFHSASKIITLKKRW<br>QLALSKGVHFVCNLLLLFVTVYSHLLLVAAGLE<br>APFLYLYALVYFLQSINFVRIIMRLWLCWKCRSK<br>NPLLYDANYFLCWHTNCYDYCIPYNSVTSSIVIT<br>SGDGTTSPISEHDYQIGGYTEKWESGVKDCVVL<br>HSYFTSDYYQLYSTQLSTDTGVEHVTFFIYNKIV<br>DEPEEHVQIHTIDGSSGVVNPVMEPIYDEPTTTTS<br>VPL*
**Gamma and descendants**	orf3a_6FY2YZ	P.1, P.1.14, P.1.17	MDLFMRIFTIGTVTLKQGEIKDATPSDFVRATATI<br>PIQASLPFGWLIVGVALLAVFQSASKIITLKKRW<br>QLALSKGVHFVCNLLLLFVTVYSHLLLVAAGLE<br>APFLYLYALVYFLQSINFVRIIMRLWLCWKCRSK<br>NPLLYDANYFLCWHTNCYDYCIPYNSVTSSIVIT<br>SGDGTTSPISEHDYQIGGYTEKWESGVKDCVVL<br>HSYFTSDYYQLYSTQLSTDTGVEHVTFFIYNKIV<br>DEPEEHVQIHTIDGSPGVVNPVMEPIYDEPTTTTS<br>VPL*
**Shared pre-VOC ORF8 lineages**	orf8_1BQFWU	AE.8, B, B.1, B.1.1, B.1.1.1, B.1.1.121, B.1.1.157, B.1.1.176, B.1.1.181, B.1.1.216, B.1.1.231, B.1.1.33, B.1.1.417, B.1.1.434, B.1.111, B.1.128, B.1.160, B.1.243, B.1.346, B.1.349, B.1.350, B.1.351, B.1.36, B.1.36.31, B.1.36.38, B.1.36.8, B.1.427, B.1.438.1, B.1.523, B.3, B.6, BA.1, BA.1.1, BA.1.1.10, BA.1.15, BA.2, BA.2.10, BA.2.3, XAP	MKFLVFLGIITTVAAFHQECSLQSCTQHQPYVVD<br>DPCPIHFYSKWYIRVGARKSAPLIELCVDEAGSK<br>SPIQYIDIGNYTVSCLPFTINCQEPKLGSLVVRCSF<br>YEDFLEYHDVRVVLDFI*
**Gamma and descendants**	orf8_SSNUW5	P.1, P.1.14, P.1.17	MKFLVFLGIITTVAAFHQECSLQSCTQHQPYVVD<br>DPCPIHFYSKWYIRVGARKSAPLIELCVDEAGSK<br>SPIQYIDIGNYTVSCLPFTINCQKPKLGSLVVRCS<br>FYEDFLEYHDVRVVLDFI*

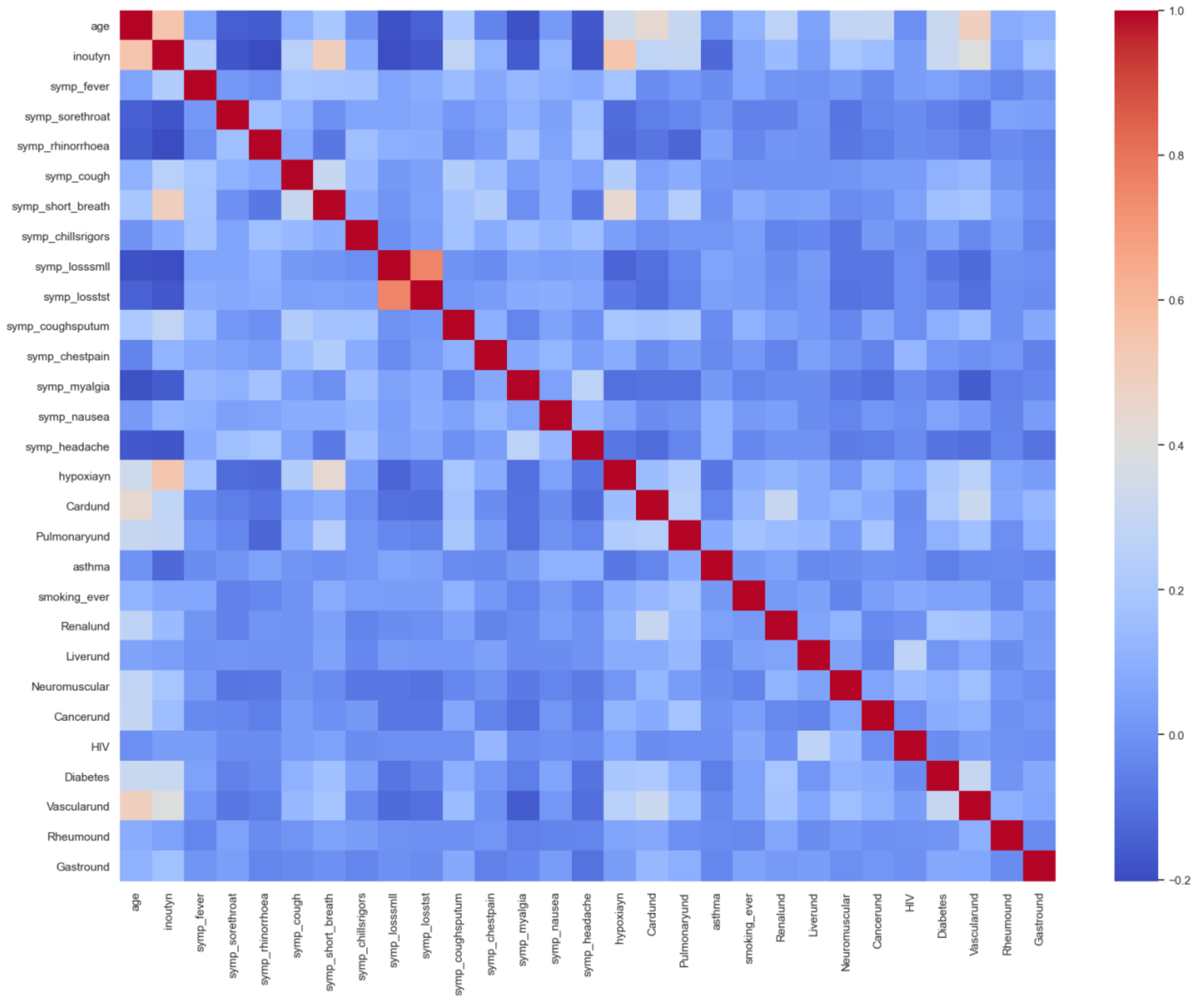

Supplementary figure 1: Correlation matrix of clinical variables.

A

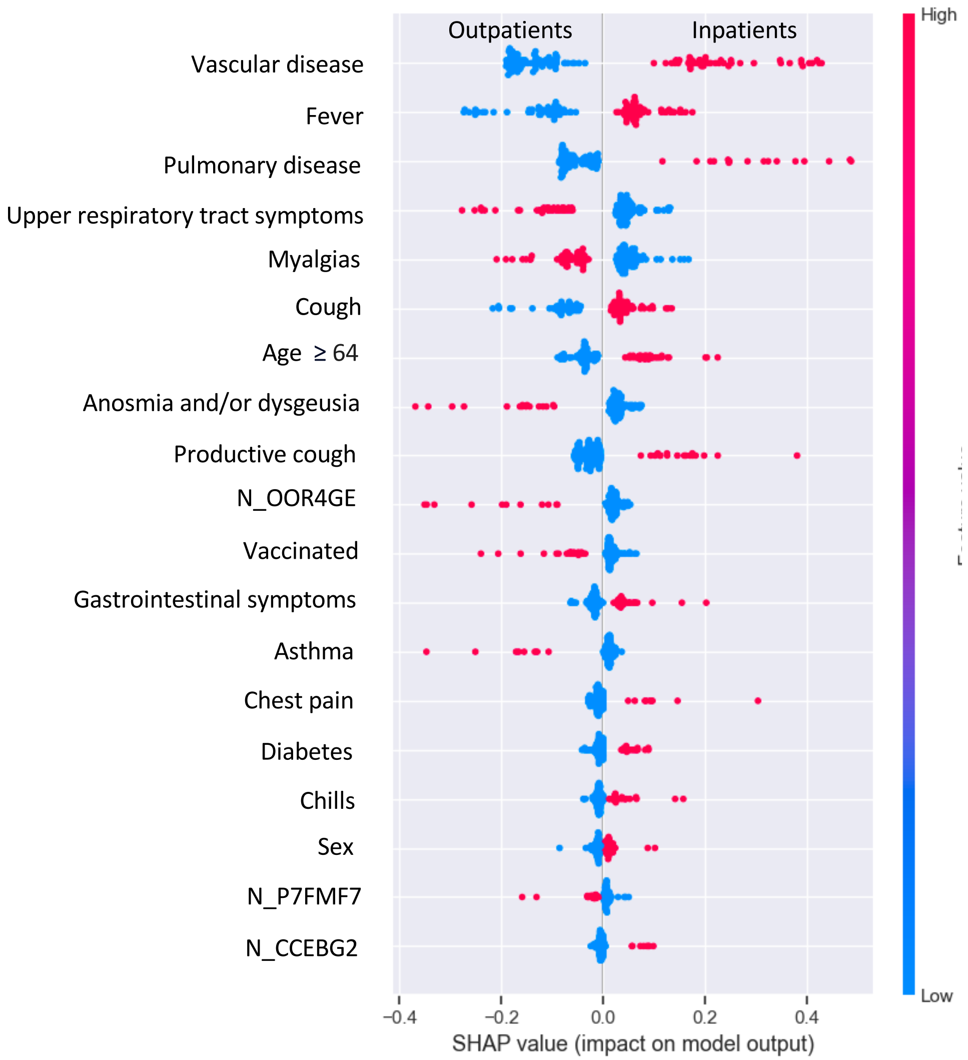

N protein

GBM + LASSO stacked model

Train recall = 0.8105 ± 0.0435

Test recall = 0.7808 ± 0.1102

Train AUROC = 0.8257 ± 0.0337

Test AUROC = 0.7923 ± 0.0504

B

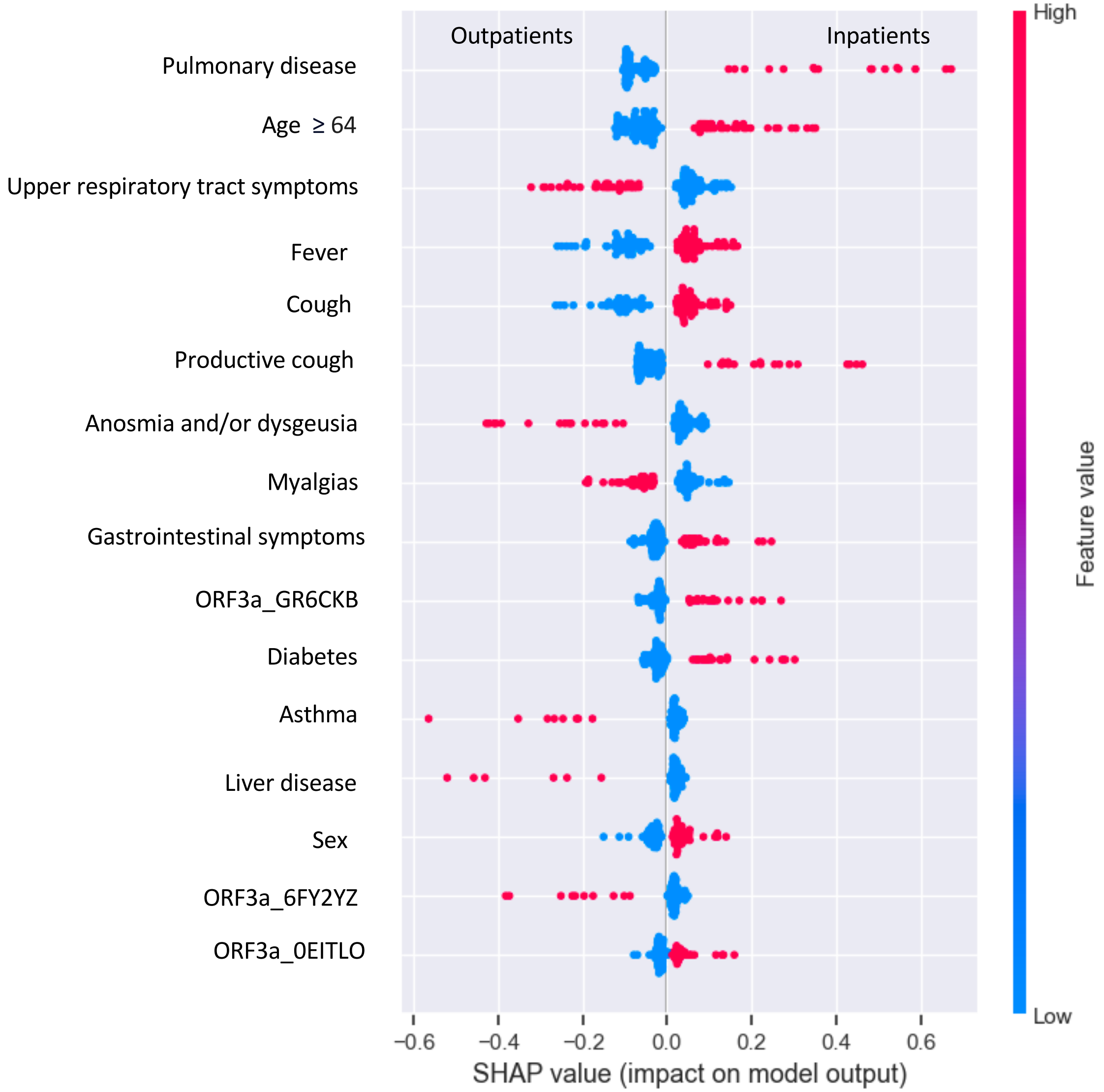

SHAP value (impact on model output)

ORF3a protein
GBM + LASSO stacked model
Train recall = 0.8224 ± 0.0437
Test recall = 0.7769 ± 0.1163
Train AUROC = 0.8296 ± 0.0379
Test AUROC = 0.7777 ± 0.809

C

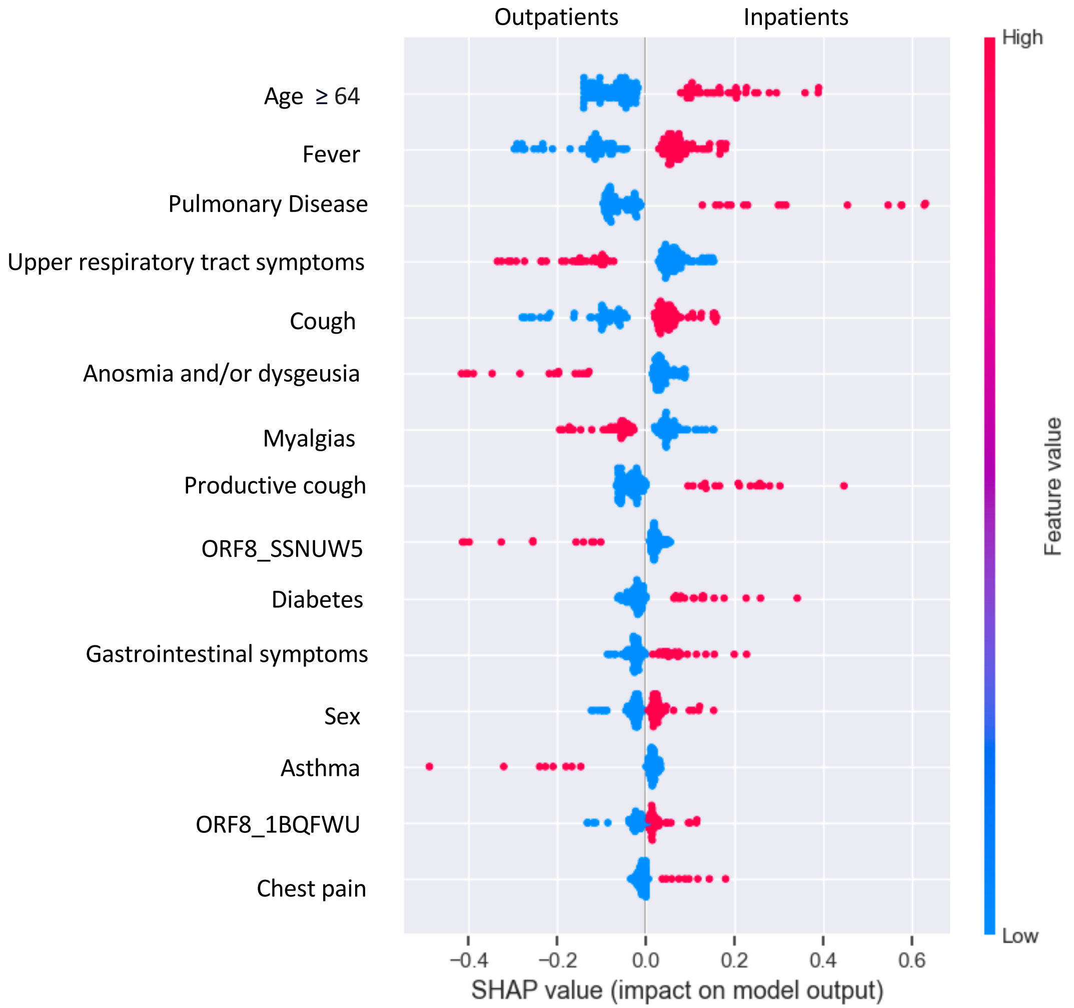

ORF8 protein

GBM + LASSO stacked model

Train recall = 0.8103 ± 0.0680

Test recall = 0.8115 ± 0.1133

Train AUROC = 0.8235 ± 0.0376

Test AUROC = 0.8162 ± 0.1133

Supplementary Figure 2: Clinical, demographic, and SARS-CoV-2 genomic features ranked in descending order of importance in predicting hospitalization for the Stacking (GBM + LR LASSO) model based on their SHapley Additive exPlanations (SHAP) values. Separate models were implemented for each of the Nucleocapsid (A), ORF3a (B), and ORF8 (C) proteins.

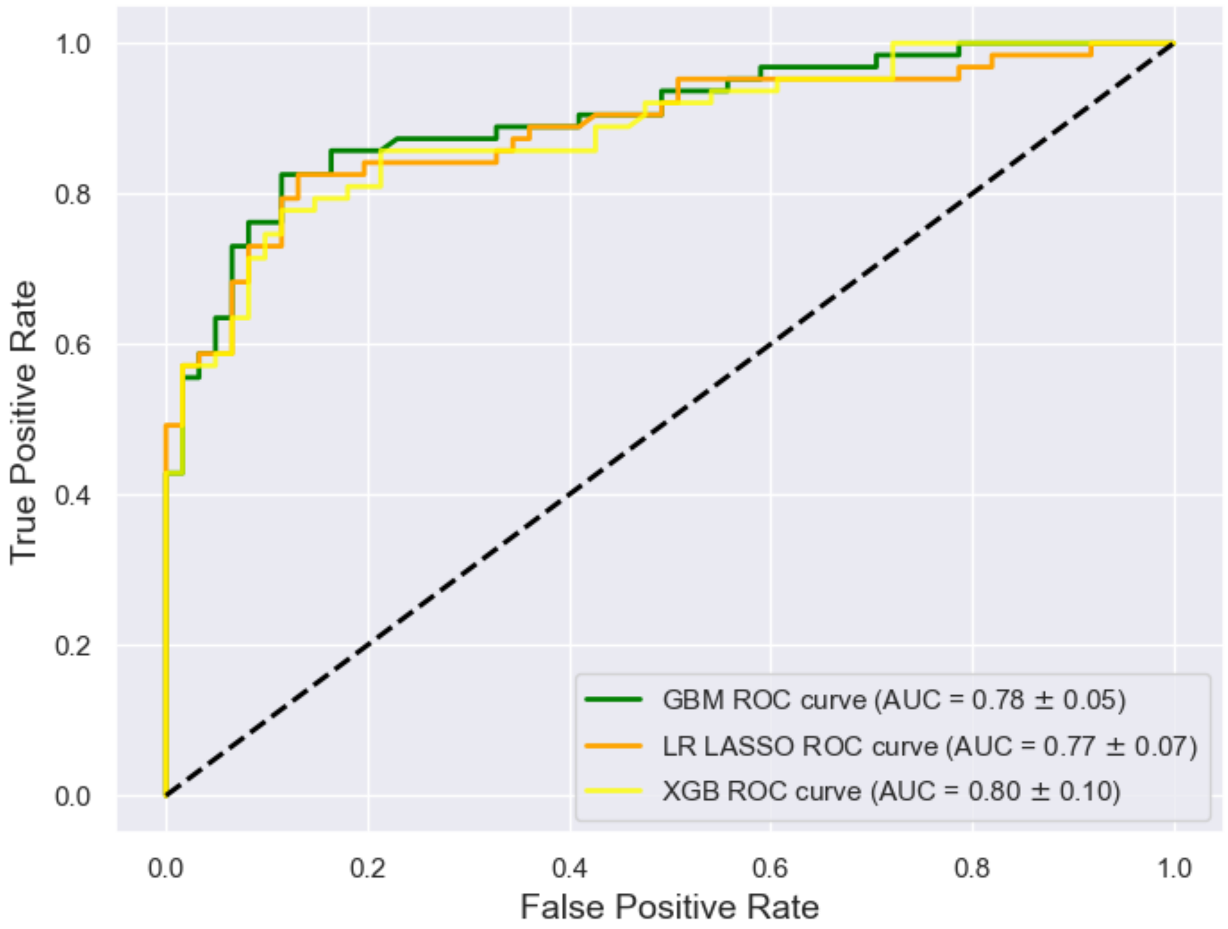

Supplementary figure 3: Receiver operating characteristic (ROC) curve for GBM, LR LASSO, and XGB models.

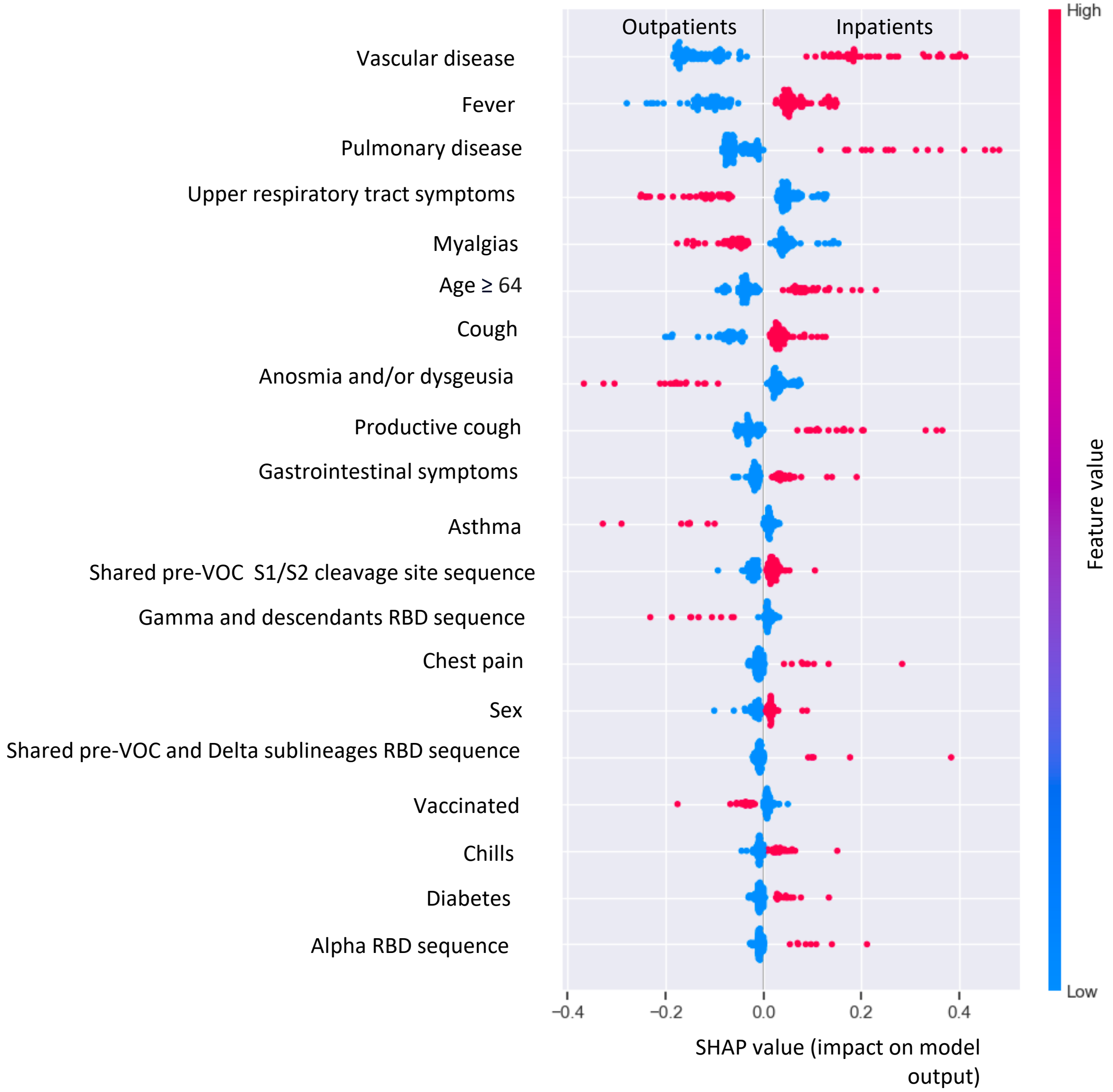

Spike protein

Stacking (GMB + LASSO) model

Train recall = 0.8107 ± 0.0514

Test recall = 0.8115 ± 0.1024

Train AUROC = 0.8339 ± 0.0484

Test AUROC = 0.8327 ± 0.0658

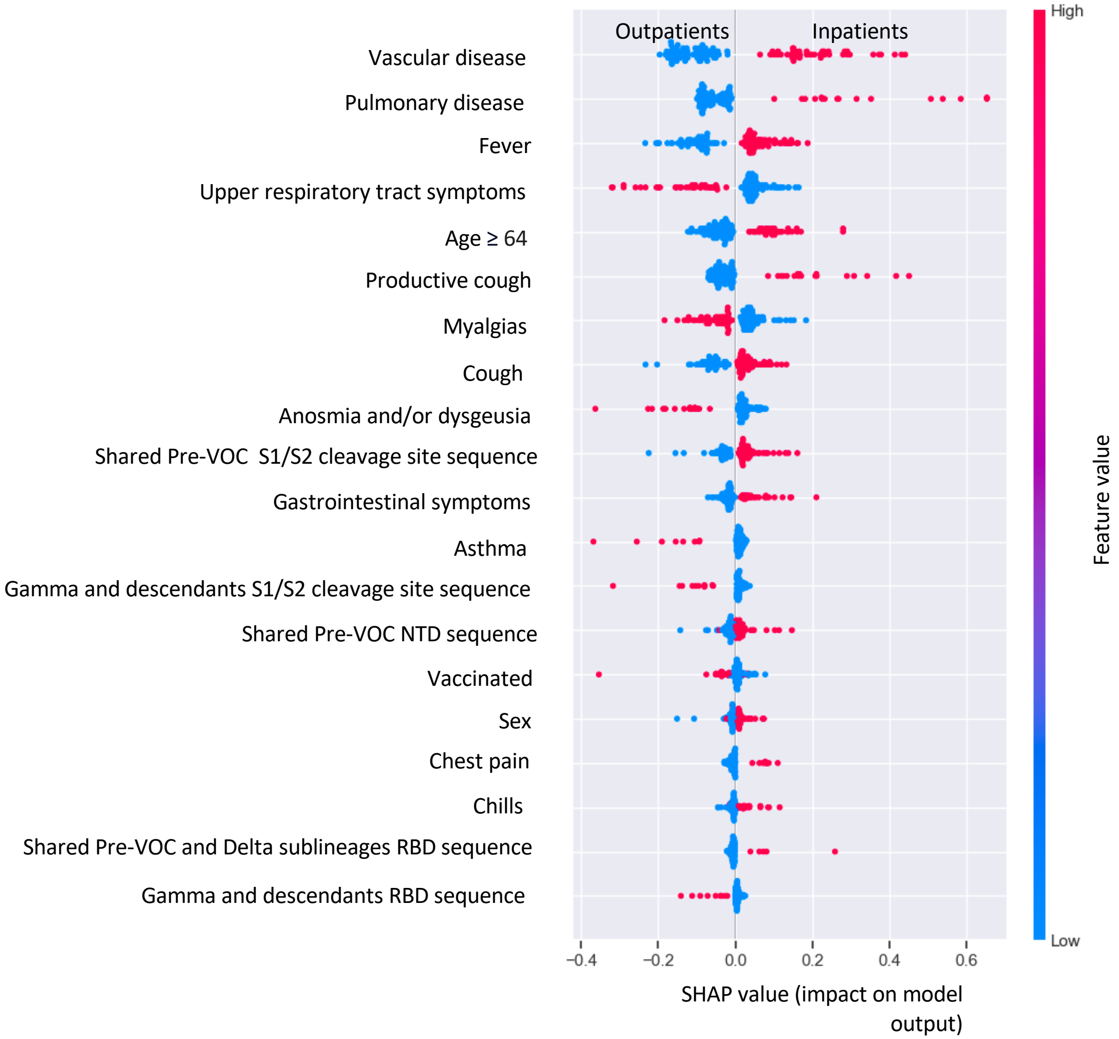

Spike protein

XGBoost model

Train recall = 0.8027 ± 0.0732

Test recall = 0.7782 ± 0.0286

Train AUROC = 0.8279 ± 0.0478

Test AUROC = 0.7974 ± 0.0954

Supplementary Figure 4: Clinical, demographic, and SARS-CoV-2 genomic features ranked in descending order of importance in predicting hospitalization for the other top performing Stacking (GBM + LASSO) (A) and XGBoost (B) models based on their SHapley Additive exPlanations (SHAP) values. Both models reflect the spike SARS-CoV-2 protein.

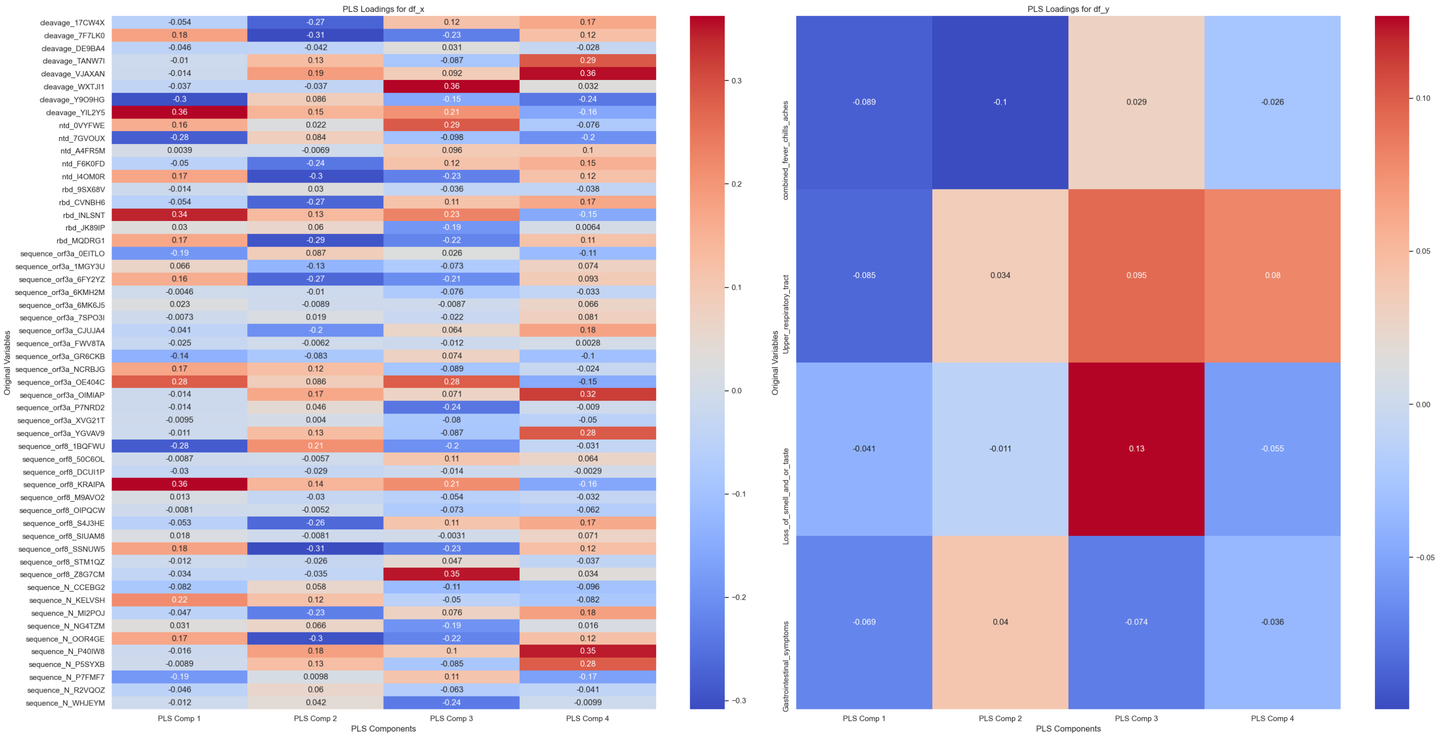

Supplementary Figure 5 : Partial Least Squares analysis of the predictor (genomic variables) and response (grouped patient symptoms) datasets.

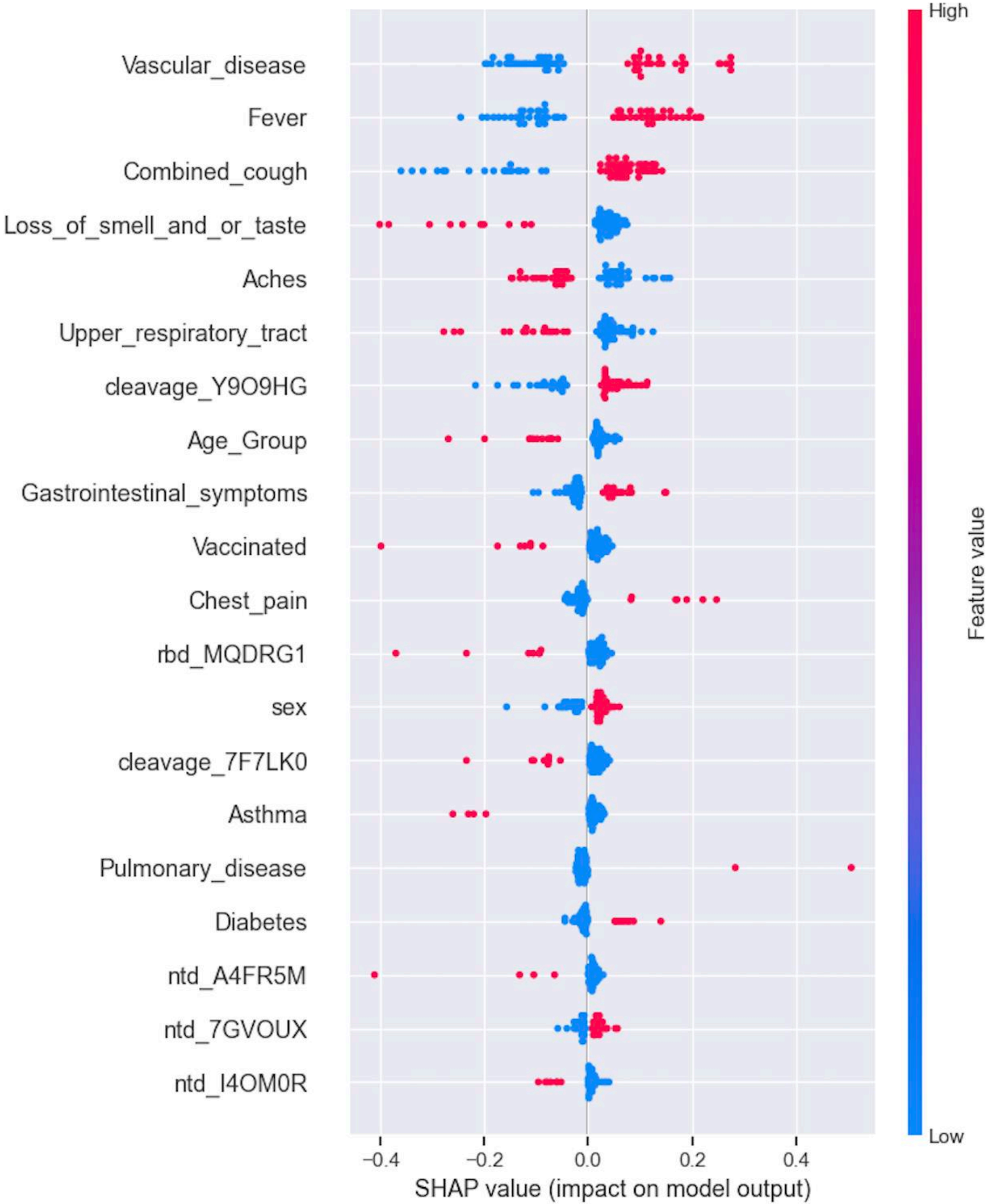

Propensity Score Matching

LR Model

Train recall: 0.7661± 0.0742

Test recall: 0.7952± 0.1441

Train AUROC: 0.7591± 0.0385

Test AUROC: 0.7000± 0.0609

Supplementary Figure 6: Clinical, demographic, and SARS-CoV-2 genomic features ranked in descending order of importance in predicting hospitalization for a logistic regression (LR) model based on SHapley Additive exPlanations (SHAP) values after performing propensity score matching (PSM) to balance age and sex. PSM aims to balance age and sex in the inpatient (treated) and outpatient (control) groups to reduce confounding. Logistic regression is used for PSM to generate propensity scores which represent the probability of being an inpatient given our covariates. Nearest-neighbour matching was used with a 1:1 ratio (one inpatient to one outpatient) to pair units with similar propensity scores to allow for comparability. Units with significantly different propensity scores that could not be matched were excluded to preserve balance. Standard mean differences were compared pre- and post- matching to evaluate the balance for each covariate using a threshold of 0.1.
